# TLS as Predictors and Targets in Neoadjuvant Chemoimmunotherapy for NSCLC

**DOI:** 10.1111/1759-7714.70375

**Published:** 2026-08-14

**Authors:** Zhiqi Li, Lina Zhang, Yujie Dong, Ling Yi, Weiying Li, Jinghui Wang, Yuanming Pan

**Affiliations:** ^1^ Cancer Research Center Beijing Chest Hospital, Capital Medical University/Beijing Tuberculosis and Thoracic Tumor Research Institute Beijing China; ^2^ Department of Pathology Beijing Chest Hospital, Capital Medical University/Beijing Tuberculosis and Thoracic Tumor Research Institute Beijing China; ^3^ School of Continuing Education, Beijing University of Chinese Medicine Beijing China

**Keywords:** immune biomarkers, neoadjuvant chemoimmunotherapy, non‐small cell lung cancer, tertiary lymphoid structures, tumor microenvironment

## Abstract

Tertiary lymphoid structures (TLS) have emerged as critical modulators of antitumor immunity in non‐small cell lung cancer (NSCLC), with growing evidence supporting their role as both prognostic biomarkers and functional targets in neoadjuvant chemoimmunotherapy. TLS are organized ectopic lymphoid aggregates that facilitate local antigen presentation, T and B cell activation, and memory immune responses within the tumor microenvironment. Their presence and maturation status correlate with favorable clinical outcomes and enhanced responsiveness to immune checkpoint inhibitors. Despite these insights, several obstacles hinder clinical translation. Current approaches remain limited in dynamically monitoring TLS evolution during treatment, and the underlying immunoregulatory mechanisms—particularly interactions among immune subsets, stromal components, and tumor cells—are not fully elucidated. Additionally, translation faces challenges including inter‐institutional variability, lack of standardized assessment criteria, and the need for multi‐center prospective validation. Integrating multimodal technologies—high‐plex imaging, spatial transcriptomics, and digital pathology—with AI‐based platforms holds promise for refined TLS characterization and real‐time monitoring. Near‐term priorities include optimizing dynamic monitoring techniques and establishing standardized scoring systems. Medium‐to‐long‐term priorities encompass multi‐center trials to validate TLS utility across diverse populations, identify novel drivers of TLS formation, and develop optimized combinatorial immunotherapy regimens that leverage TLS as both predictive biomarkers and therapeutic targets. By bridging basic immunology and clinical oncology, TLS are poised to emerge as key regulatory nodes in precision cancer immunotherapy, which may catalyze a paradigm shift in the neoadjuvant NSCLC landscape, enabling a closed‐loop management framework encompassing prediction, intervention, and dynamic efficacy optimization across the entire continuum of patient care.

## Graphical abstract

TLS in lung cancer adjuvant therapy: (A) cellular composition (T/B cells, DCs); (B) mechanistic pathways (antigen presentation, GC‐like B responses, TRM maintenance); (C) clinical evaluation (IHC, digital pathology, spatial transcriptomics); (D) future directions (dynamic monitoring, intercellular interactions, multicenter trials, clinical translation).

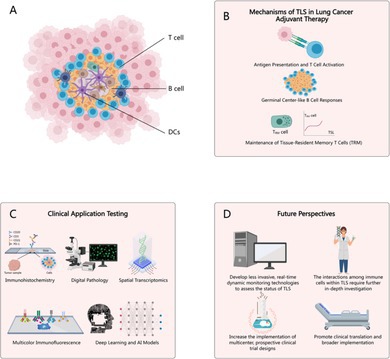

**Abbreviations**
BCR: B‐cell receptor
CAF: cancer‐associated fibroblast
CRC: colorectal cancer
CTL: cytotoxic T lymphocyte
CTLA‐4: cytotoxic T lymphocyte‐associated protein‐4
DC: dendritic cell
DFS: disease‐free survival
ECM: extracellular matrix
EFS: event‐free survival
EGFR: epidermal growth factor receptor
FDC: follicular dendritic cell
GC: germinal center
GITR: glucocorticoid‐induced tumor necrosis factor receptor‐related protein
HCC: hepatocellular carcinoma
H&E: hematoxylin and eosin
HEV: high endothelial venule
HNSCC: head and neck squamous cell carcinoma
ICI: immune checkpoint inhibitor
IHC: immunohistochemistry
LDRT: low‐dose radiotherapy
MDSC: myeloid‐derived suppressor cell
MHC: major histocompatibility complex
mIF: multiplex immunofluorescence
MPR: major pathological response
NACT: neoadjuvant chemotherapy
NSCLC: non‐small cell lung cancer
OS: overall survival
PC: plasma cell
pCR: pathological complete response
PD‐1: programmed cell death protein 1
PD‐L1: programmed death‐ligand 1
PFS: progression‐free survival
PLR: platelet‐lymphocyte ratio
RAITS: radiomics integrated TLS system
RCC: renal cell carcinoma
SCC: squamous cell carcinoma
scRNA‐seq: single‐cell RNA sequencing
SHM: somatic hypermutation
SII: systemic immune‐inflammation index
STAS: spread through air spaces
TAM: tumor‐associated macrophage
TCR: T‐cell receptor
Tfh: T follicular helper cell
TLS: tertiary lymphoid structure
TME: tumor microenvironment
Treg: regulatory T cell
Trm: tissue‐resident memory T cell
UMI: unique molecular identifier
WGCNA: weighted gene co‐expression network analysis

## 1 Introduction

### 1.1 Research Background and Historical Evolution

**FIGURE 1:**
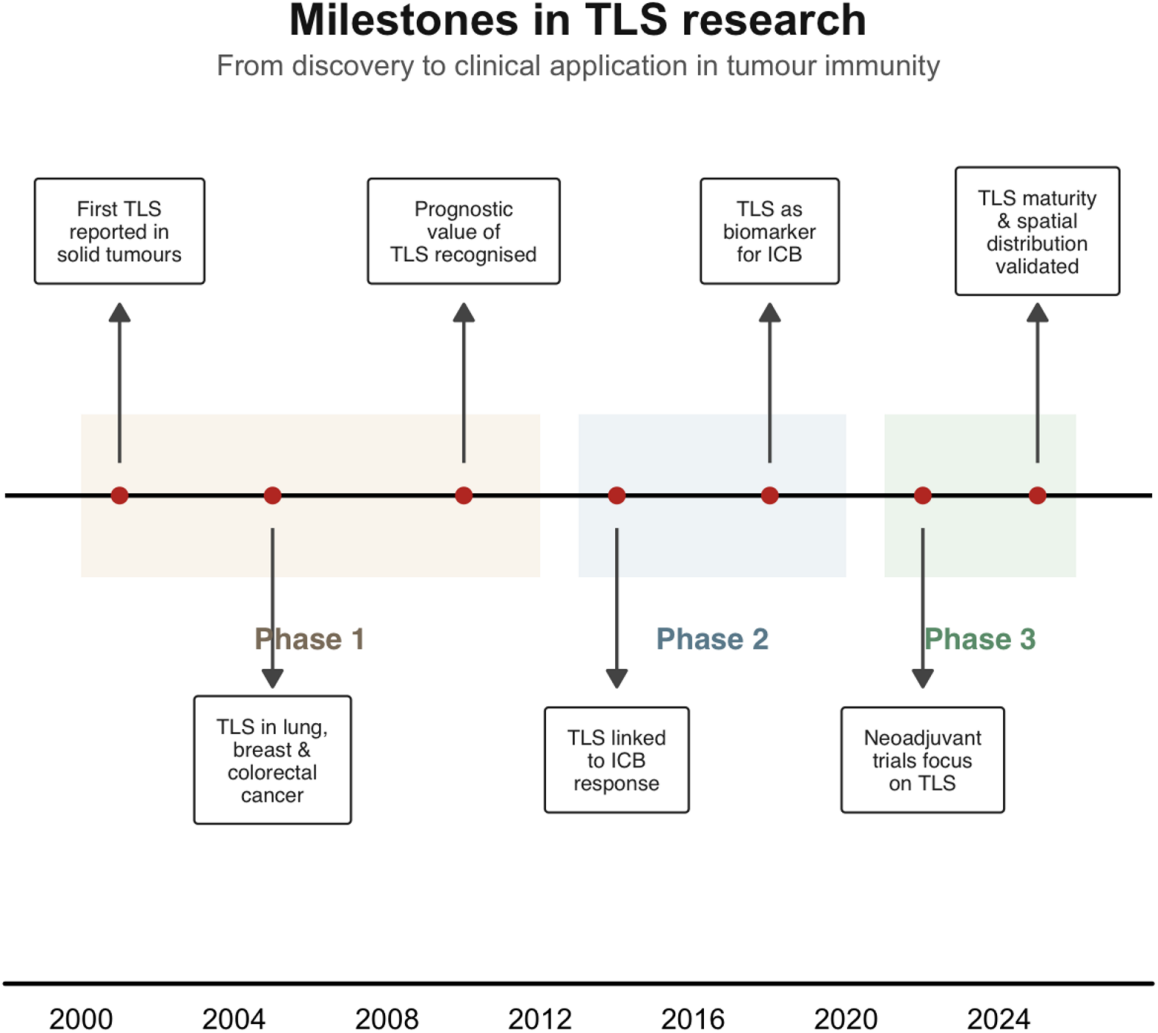
Timeline of TLS research in cancer immunity. Milestones spanning 2000–2026 are shown along a horizontal axis, with three shaded phases representing sequential waves of discovery: Initial tumor‐associated TLS reports (Phase 1), association with ICB response (Phase 2), and validation of TLS features as biomarkers in neoadjuvant settings (Phase 3). ICB, immune checkpoint blockade; TLS, tertiary lymphoid structure.

**FIGURE 2:**
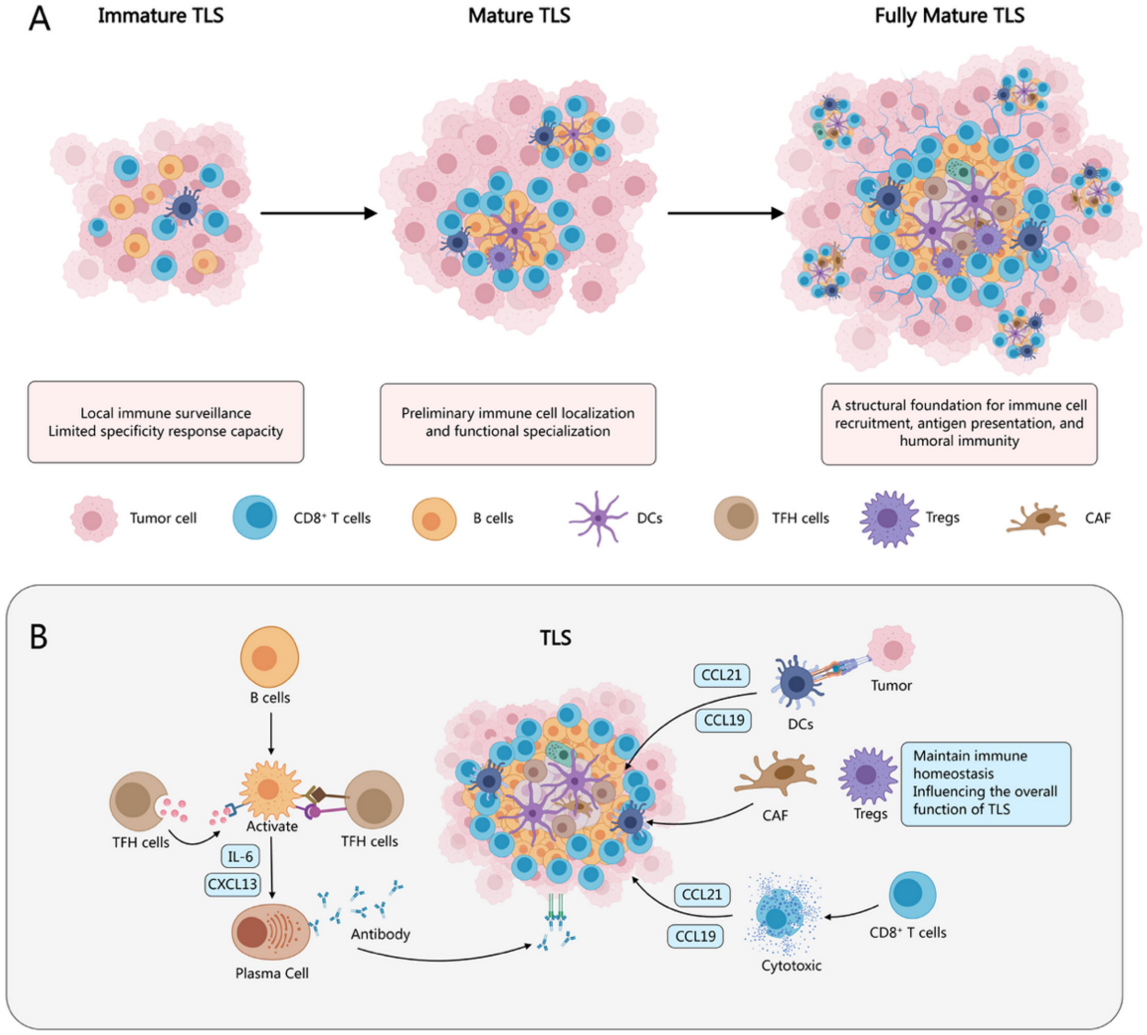
Morphological maturation stages of tertiary lymphoid structures (TLS) and their functional implications. (A) Immature TLS. This early stage is characterized by limited structural organization and modest cellular composition, enabling local immune surveillance but with restricted specificity and responsiveness. Key cellular components include tumor cells, CD8^+^ T cells, B cells, dendritic cells (DCs), T follicular helper (TFH) cells, regulatory T cells (Tregs), and cancer‐associated fibroblasts (CAFs). Mature TLS. Progressive structural maturation leads to a more organized architecture with segregated T‐ and B‐cell zones, supporting robust adaptive immune responses. The coordinated cellular microenvironment maintains immune homeostasis and modulates TLS functionality. Fully mature TLS. At this terminal stage, fully mature TLS provide a well‐defined structural foundation for efficient immune cell recruitment, antigen presentation, and humoral immunity, correlating with enhanced anti‐tumor immunity and favorable clinical outcomes. (B) At the most advanced stage, fully mature TLS exhibit well‐defined structural frameworks that facilitate efficient immune cell recruitment, antigen presentation, and humoral immunity. These organized lymphoid aggregates are thought to correlate with improved anti‐tumor immune responses and favorable clinical outcomes in the context of cancer immunotherapy and adjuvant therapy.

Research on the tumor microenvironment (TME) began in the late 20th century, and the phenomenon of lymphoid structures arising in non‐lymphoid tissues soon attracted keen interest [1]. Early studies focused mainly on ectopic lymphoid structures in autoimmune diseases and chronic infections [2, 3]. For example, lymphocyte clusters were observed in chronic inflammatory lesions of rheumatoid arthritis and chronic obstructive pulmonary disease, where they induced histological changes resembling those of secondary lymphoid organs [4, 5]. As research deepened, this phenomenon was gradually recognized in tumor tissues, offering a new perspective on local immune responses [6]. The term “tertiary lymphoid structures (TLS)” emerged to distinguish them from congenital primary (e.g., thymus) and secondary (e.g., lymph nodes, spleen) lymphoid organs [1] (Figure 1). TLS are structures that form spontaneously in non‐lymphoid tissues through lymphoid neogenesis driven by persistent antigen stimulation or chronic inflammation (Figure 2A) [7, 8]. Their typical morphology includes: high endothelial venules (HEVs) at the periphery that efficiently recruit circulating lymphocytes; relatively independent B‐cell follicles and T‐cell zones inside; and areas rich in mature dendritic cells, as visualized by immunohistochemistry or multi‐color immunofluorescence staining [9, 10]. In some mature TLS, a germinal center‐like process of B‐cell proliferation and affinity maturation even occurs [11]. Because they provide a microenvironment for antigen presentation and T/B cell activation and proliferation, TLS are regarded as important hubs for local immune responses, not merely as passive inflammatory reactions (Figure 2B) [12, 13].

In lung cancer tissues, scattered or aggregated lymphoid nodules were first observed with H&E staining; later, immunohistochemical techniques revealed the spatial distribution and interaction trends of key components such as B cells (CD20^+^), T cells (CD3^+^), and dendritic cells (DC‐LAMP^+^) [6, 14]. For example, a retrospective histological analysis of patients receiving neoadjuvant chemotherapy showed that TLS were widely present in resected NSCLC specimens, and their abundance was closely related to postoperative survival [15]. This discovery marked the beginning of clinical attention to the practical value of TLS in lung cancer research.

In the genomic era, multi‐omic profiling has been extensively applied to tumor tissue analysis [16, 17]. Multi‐omic profiling has revealed distinct states of TLS in the TME. Activated TLS are associated with improved prognosis, whereas a hypoxic microenvironment appears to suppress TLS development and is linked to poor prognosis [7, 18]. These findings not only confirm the structural characteristics of TLS but also provide multi‐dimensional data supporting its functional positioning.

From a historical perspective, the evolution of TLS research in tumor immunity can be divided into three stages: early 2000s‐TLS were successively reported in various solid tumors, including lung cancer, breast cancer, and colorectal cancer [19, 20, 21]; mid 2010s—with the rise of immune checkpoint inhibitors (ICIs), researchers found that TLS abundance was positively correlated with ICI efficacy, further fueling research into TLS [12, 13]; past 5 years‐clinical studies based on neoadjuvant immunotherapy and chemoimmunotherapy have increasingly focused on the quantity, maturity, and spatial distribution of TLS after surgery, and their value in evaluating treatment response and predicting prognosis has been widely validated [22, 23]. Overall, TLS research has progressed from “phenomenon description” to “mechanism analysis” and then to “clinical translation.” Their central role in local tumor immune regulation has become increasingly clear, offering new ideas for optimizing lung cancer immunotherapy strategies [24, 25].

### 1.2 Neoadjuvant Treatment of NSCLC: Challenges and Roles of TLS in Tumor Immunity

Non‐small cell lung cancer (NSCLC) is one of the most common and deadly malignant tumors globally, accounting for approximately 80%–85% of all lung cancer cases [26]. Surgical resection remains the mainstay of treatment for early‐stage patients, but recurrence occurs in up to 55% of cases even after complete resection, primarily due to occult metastatic disease [27]. In recent years, neoadjuvant treatment—including preoperative chemotherapy, neoadjuvant immunotherapy, and targeted therapy—has been widely incorporated into multicenter clinical trials and routine practice, with the goals of reducing tumor burden, eliminating micro‐metastases, and improving pathological response rates before surgery [28]. However, clinical data reveal marked heterogeneity in patients' responses to neoadjuvant strategies: in PD‐1/PD‐L1 inhibitor plus chemotherapy regimens, the major pathologic response rate ranges from 30% to 60%, the pathologic complete response rate from 17% to 18%, and approximately 40%–60% of patients fail to achieve durable benefits, underscoring the urgent need for reliable biomarkers to guide individualized treatment decisions [29, 30, 31].

Neoadjuvant intervention not only exerts direct cytotoxic effects, but also reshapes the immune landscape of the tumor microenvironment (TME) [32]. As the “in situ immune command center” within tumors, tertiary lymphoid structures (TLS)—ectopic lymphoid aggregates that develop in non‐lymphoid tissues and are linked to better prognosis and stronger antitumor immune responses in NSCLC—play a pivotal role in orchestrating local adaptive immune responses [6, 7].

1. Antigen presentation and T‐cell activation: High endothelial venules (HEVs), specialized postcapillary venules that serve to recruit circulating lymphocytes to secondary lymphoid organs and have been described in both human cancer and chronic inflammatory diseases, mediate the infiltration of large numbers of circulating lymphocytes into tumors [9, 20]. In TLS, dendritic cells take up antigens and present them to T cells, enabling tumor antigen presentation and triggering initial immune responses [10]. Mature TLS further facilitate major histocompatibility complex (MHC) antigen processing and presentation, enhancing the priming of effector T cells, while TLS‐associated B cells also play a major role in antigen presentation and T‐cell polarization [24].
2. B‐cell germinal center‐like reaction: Mature TLS contain follicular dendritic cells (FDCs) and support B‐cell differentiation into antibody‐producing plasma cells (PCs) via germinal center (GC) reactions, where B cells undergo clonal expansion, somatic hypermutation, and isotype switching, generating tumor‐reactive antibodies (IgG, IgA) [11, 12]. In patients achieving pathologic complete response (pCR) after neoadjuvant chemoimmunotherapy, TLS‐associated regions display higher plasma cell content, whereas non‐responding tumors contain more naïve B cells, indicating that functional humoral responses within TLS are critical for treatment success [13]. Emerging evidence also suggests that the quality of TLS—such as the degree of maturation and the nature of infiltrating immune cells—may further influence immunotherapeutic outcomes [33].
3. Maintenance of tissue‐resident memory T cells (Trm): The TLS microenvironment supports the persistent activation and maintenance of CD4^+^, CD8^+^CD103^+^ Trm cells, making long‐term anti‐tumor immune surveillance possible [34]. In breast cancer, CD103^+^ CD8^+^ Trm cells within tumor‐associated TLS contribute to anti‐tumor immune responses, and the effector function of CD8^+^ CD103^+^ Trm cells is strongly correlated with TLS maturity [35]. Tissue‐resident immune cells, including Trm cells, are emerging as potential key players in tumor infiltration and TLS formation due to their ability to reside indefinitely within tissues and mount effective responses to local antigens [36].

Numerous evidence‐based studies have confirmed that TLS abundance, maturity, and spatial distribution are highly correlated with ICIs efficacy [7, 10]. A comprehensive meta‐analysis of 19 clinical trials including 1752 patients demonstrated that high TLS density is associated with a significantly increased response rate to ICIs (OR = 2.99, 95% CI: 2.14–4.18, *p* < 0.001) and prolonged progression‐free survival (HR = 0.75, 95% CI: 0.63–0.88, *p* < 0.001) [37]. In NSCLC specifically, this trend has been validated, with TLS abundance positively correlated with response to PD‐1/PD‐L1 inhibitors, making TLS a promising predictive biomarker for immunotherapy [23, 38].

It must be noted, however, that TLS function is not uniformly beneficial. Immature or dysfunctional TLS may be enriched with regulatory T cells (Tregs) and inhibitory fibroblasts, forming an immunosuppressive microenvironment [39, 40]. TLS exhibit a bifunctional nature: their spatial organization and dynamic interactions with stromal elements dictate the balance between immune activation and tolerance within the TME [24, 41]. Thus, understanding the regulation of TLS plasticity is essential for developing effective combination treatment strategies [1].

From a clinical translation perspective, TLS hold several important applications: (i) Biomarker value: Integrating TLS‐related indicators, such as density, maturity, and spatial distribution, can refine predictive models for neoadjuvant efficacy, facilitating the quantification of biomarkers for precision medicine [7, 42]. (ii) Therapeutic targeting: Elucidating the regulatory mechanisms of TLS provides theoretical support for novel combination regimens. For example, modulating the chemokine CXCL13, which is critical for TLS formation and function, has shown promise: CXCL13 agonists combined with anti‐PD‐1 therapy have been shown to enhance antitumor activity in several cancer models, and radiotherapy combined with PD‐L1 blockade significantly increases CXCL13^+^ T cells within tumors [12, 43]. These observations position TLS as an actionable target for therapeutic intervention [13].

Overall, as a lymph node‐like immune microenvironment structure in local tumor tissues, TLS possess the dual functions of antigen presentation, effector cell activation, and memory maintenance. Their central role in neoadjuvant treatment of lung cancer has been increasingly established, providing important insights for further understanding tumor immune responses and optimizing individualized therapeutic strategies [6, 23].

## 2 Biological Characteristics and Formation Mechanism of TLS

### 2.1 Composition, Classification, and Maturity Stratification of TLS

The basic cellular composition of TLS: B cells, CD4^+^CXCR5^+^ T follicular helper cells, CD8^+^ cytotoxic T cells, mature dendritic cells, and variable numbers of regulatory T cells (Tregs) and cancer‐associated fibroblasts (CAFs) is well established in the literature and will not be reiterated in detail here [7, 44]. Instead, this section focuses on features that are specific to or particularly relevant in the context of neoadjuvant chemoimmunotherapy for NSCLC.

#### 2.1.1 Pre‐Existing Versus Therapy‐Induced TLS: Distinct Origins and Potential Functional Differences

TLS observed in post‐treatment resection specimens can originate from two scenarios: (i) Pre‐existing TLS (present before neoadjuvant therapy): These structures represent a baseline immune hub. Their abundance and maturity may predict inherent sensitivity to immunotherapy [42, 45]. Baseline immune activation, marked by higher infiltration of CD20^+^ B cells and cytotoxic T lymphocytes, has been shown to correlate with achieving major pathologic response (MPR) or pathologic complete response (pCR) [46]. However, neoadjuvant therapy can further remodel them, for example, by increasing the CD8^+^/Treg ratio or enhancing Tfh–B cell interactions [47]. (ii) Therapy‐induced TLS (de novo formed during neoadjuvant treatment): Chemotherapy and/or immune checkpoint inhibitors can drive de novo TLS neogenesis or accelerate the maturation of previously immature aggregates [48]. Patients who undergo neoadjuvant chemotherapy or immunochemotherapy exhibit increased TLS density compared to those who opt for upfront surgery [12]. Preliminary evidence suggests that therapy‐induced TLS may exhibit higher expression of homeostatic chemokines (CXCL13, CCL19, CCL21) and a more favorable cytotoxic‐to‐regulatory cell balance compared to their pre‐existing counterparts [7, 49].

Critically, most published studies have not distinguished between these two populations, as baseline tumor tissue is rarely available [46]. This represents a major knowledge gap. Clarifying whether pre‐existing versus therapy‐induced TLS carry different prognostic or predictive value is essential for biomarker development and for designing strategies that intentionally foster TLS formation [50].

#### 2.1.2 Maturity Stratification in the Neoadjuvant Setting

While TLS maturity (immature aggregates → primary follicles → fully mature TLS with germinal centers) follows a common histological continuum, its clinical implications are context dependent. In neoadjuvant‐treated NSCLC: (i) Fully mature TLS in post‐treatment resected specimens are consistently associated with major pathological response (MPR), pathological complete response (pCR), and prolonged progression‐free survival [7, 42]. A double‐center retrospective study of 80 patients with resectable NSCLC (stage IB‐IIIB) receiving neoadjuvant chemoimmunotherapy reported that patients with high TLS maturation had significantly longer disease‐free survival compared to those with low TLS maturation (median DFS: 34.07 months vs. 22.30 months, *p* = 0.024), and TLS high‐maturation was identified as a prognostic factor [51]. Additionally, high expression and maturation of TLS were significantly associated with improved clinical outcomes across most subgroups [51]. Beyond NSCLC, neoadjuvant chemoimmunotherapy has also been demonstrated to promote mature TLS formation in head and neck squamous cell carcinoma, where TLS maturity correlated with favorable immunotherapy response [52]. (ii) Immature TLS may represent either a “pre‐active” state that can be therapeutically matured or a dysfunctional, potentially immunosuppressive niche (e.g., when enriched with regulatory T cells, Tregs) [53]. In NSCLC, Tregs infiltrating tumor‐induced TLS have been shown to inhibit the proliferation and cytokine secretion of CD4^+^ conventional T cells, and the presence of Tregs within TLS is associated with poor clinical outcome [22]. The cellular composition within immature TLS differs from that of mature TLS: single‐cell and spatial transcriptomics studies have revealed that immature TLSs are enriched in FCRL4^+^ B cells and peripheral helper T cells, whereas mature TLSs contain germinal center B cells, follicular helper T cells, and resident memory CD8^+^ T cells [52].

The dynamic plasticity of TLS under therapy, that is, the possibility of converting immature aggregates into functional, mature structures, is a unique feature of the neoadjuvant setting that is not typically observed in autoimmune or chronic infectious diseases [54]. Neoadjuvant immunotherapy combined with chemotherapy has been demonstrated to promote TLS formation and enhance immune cell populations, including CD3^+^ T cells and CD8^+^ T cells, which is associated with improved survival outcomes in resectable NSCLC [46, 48]. Moreover, neoadjuvant therapies can remodel pre‐existing TLS by increasing the CD8^+^/Treg ratio and enhancing Tfh‐B cell interactions [55]. Understanding this therapy‐induced TLS maturation process is essential for developing strategies that intentionally foster functional TLS formation to improve immunotherapy efficacy.

#### 2.1.3 Comparison With TLS in Other Contexts

Although TLS share core histological features across diseases, their functional outcomes differ markedly (Table 1). This contextual divergence underscores the critical importance of understanding TLS biology within the specific disease milieu rather than generalizing findings across disparate conditions.

**TABLE 1:** TLS across disease contexts: A functional comparison.

Context	Functional roles	Dynamic under therapy
Autoimmune (e.g., rheumatoid arthritis) [56]	Persistent tissue destruction, autoantibody production	Generally refractory to therapy; chronicity
Chronic infection (e.g., hepatitis C) [57]	Pathogen containment, but may lead to fibrosis or lymphoma	Slow evolution; not pharmacologically inducible for resolution
Neoadjuvant NSCLC [51, 58]	Therapy‐augmented antitumor immunity; favorable prognosis	Time‐limited neogenesis (peaks after 2–3 cycles); maturation can be enhanced by chemoimmunotherapy

In autoimmune diseases such as rheumatoid arthritis, TLS serve as sites of persistent tissue destruction and autoantibody production. These structures are generally refractory to therapeutic intervention and contribute to disease chronicity, reflecting their role as drivers of pathological inflammation rather than protective immunity. The persistence of TLS in autoimmune settings is supported by sustained autoantigen exposure and dysregulated immune checkpoints, rendering them difficult to reverse pharmacologically [56].

In chronic infections such as hepatitis C, TLS function primarily in pathogen containment, providing a localized immune response that limits viral spread. However, chronic antigen stimulation within these structures may paradoxically lead to adverse outcomes, including tissue fibrosis or lymphomagenesis [57]. The evolution of TLS in chronic infection is notably slow, and unlike in the neoadjuvant cancer setting, these structures are not readily inducible for resolution through pharmacological intervention.

The neoadjuvant NSCLC setting confers unique characteristics that distinguish TLS in cancer from those in autoimmune or infectious contexts. In NSCLC, TLS are associated with therapy‐augmented antitumor immunity and favorable prognosis, representing an adaptive immune response that can be therapeutically harnessed. Critically, TLS in the neoadjuvant setting exhibit time‐limited neogenesis, peaking after 2–3 cycles of chemoimmunotherapy, and their maturation can be enhanced by combined treatment regimens. This temporal plasticity and pharmacologic inducibility are distinctive features not typically observed in autoimmune or chronic infectious diseases [51, 58]. The capacity to actively induce and mature TLS through therapeutic intervention underscores their potential as both predictive biomarkers and actionable targets in cancer immunotherapy.

Thus, while the “blueprint” of TLS is conserved, the neoadjuvant cancer setting confers unique features: (i) TLS can be therapeutically induced or matured within weeks [50]; (ii) their presence correlates with treatment efficacy rather than disease chronification [50]; and (iii) they represent a dynamic, pharmacologically targetable biomarker rather than a static lesion [59]. Understanding these context‐dependent functional differences is essential for translating TLS biology into clinical applications. While targeting TLS in autoimmune diseases would aim to suppress pathogenic lymphoid neogenesis, strategies in oncology focus on promoting TLS formation and maturation to enhance antitumor immunity. This dichotomy highlights the need for disease‐specific approaches to TLS modulation and emphasizes the importance of contextualizing TLS research findings within their appropriate pathological framework.

### 2.2 Differential Effects of Neoadjuvant Regimens on TLS and the Predictive Value of Baseline Versus Therapy‐Induced TLS for pCR

Emerging evidence indicates that both neoadjuvant chemotherapy alone and chemoimmunotherapy can increase the density and maturity of TLS in resected NSCLC specimens compared to upfront surgery [60]. However, the magnitude and functional consequences differ between the two modalities.

#### 2.2.1 Chemoimmunotherapy Versus Chemotherapy Alone: Distinct TLS Remodeling Profiles

Several comparative studies have addressed this issue. Wang et al. [46] reported that neoadjuvant immunochemotherapy (IO‐CT) not only preserved but also enhanced intratumoral immune cell populations, with notable TLS formation that correlated with improved survival outcomes. In contrast, chemotherapy alone induced a more limited and less sustained TLS maturation response. Similarly, Jiang et al. [23] confirmed that both neoadjuvant chemotherapy (NACT) and immunochemotherapy (NACT‐IO) significantly increased TLS density compared to untreated surgical controls; however, the TLS area‐to‐tumor ratio was capable of distinguishing responders from non‐responders only in the immunochemotherapy group, suggesting that TLS functionality is more intimately linked to the addition of immune checkpoint inhibitors.

In a study focusing on locally advanced lung squamous cell carcinoma, Jiang et al. [23] demonstrated that while NACT alone increased total TLS count and the proportion of germinal center (GC)‐positive TLS, the combination with immunotherapy (NACT‐IO) produced a significantly stronger effect (*p* < 0.001 for NACT‐IO vs. *p* < 0.01 for NACT alone). Furthermore, the authors observed an inverted U‐shaped correlation between GC‐positive TLS density and the number of treatment cycles, with peak maturation occurring after ≤ 2 cycles, followed by a decline thereafter. This temporal pattern was more pronounced in the combination therapy arm, indicating that chemoimmunotherapy not only induces TLS neogenesis but also accelerates and optimizes their functional maturation within a specific therapeutic window [23].

Collectively, these data indicate that although chemotherapy alone possesses some capacity to induce TLS formation, the addition of immunotherapy amplifies TLS maturation, enriches for functional GC reactions, and generates a more robust antitumor immune microenvironment.

#### 2.2.2 Baseline TLS Versus Treatment‐Induced TLS: Predictive Value for pCR

A critical unanswered question is whether pre‐existing TLS (present before treatment) are responsible for treatment sensitivity, or whether the sensitivity is driven by TLS that are induced de novo during neoadjuvant therapy.

Available evidence supports a dual contribution. Regarding baseline TLS, Wang et al. [46] found that patients who achieved major pathological response (MPR) or pCR had significantly higher pre‐treatment infiltration of CD20^+^ B cells and cytotoxic T lymphocytes (CTLs) within tumor tissues, suggesting that a pre‐existing immune‐active microenvironment, including baseline TLS, predicts inherent sensitivity to subsequent chemoimmunotherapy. Moreover, a prospective study of stage IIIA NSCLC patients receiving neoadjuvant chemoimmunotherapy showed that larger TLS areas in pre‐treatment biopsies correlated with improved event‐free survival (EFS), underscoring that baseline TLS abundance itself has independent prognostic value [29].

On the other hand, therapy‐induced TLS also play an indispensable role. Jiang et al. [23] clearly demonstrated that neoadjuvant treatment (both NACT and NACT‐IO) induces multidimensional dynamic remodeling of TLS, including increased abundance, spatial redistribution toward the invasive margin, and enhanced maturity. Notably, patients who achieved pCR exhibited a higher density of mature TLS in the post‐treatment tumor bed, and the change in TLS characteristics from baseline (when paired biopsies were available) was more predictive of pCR than baseline TLS density alone [23].

Thus, the current literature suggests that both baseline and treatment‐induced TLS carry predictive weight, but their relative importance may differ according to the endpoint. Baseline TLS abundance appears to reflect intrinsic immune fitness and is associated with a higher likelihood of response even before treatment begins [29]. Simultaneously, the dynamic maturation of TLS during therapy—particularly the emergence of GC‐positive structures within the first 2 cycles—is a strong on‐treatment predictor of pCR [23]. At present, direct comparisons using paired pre‐ and post‐treatment biopsies are limited, and no study has definitively separated the two effects. Therefore, we propose that future prospective trials should systematically collect both baseline (biopsy) and post‐treatment (resection) tissue to quantify the incremental predictive value of TLS neogenesis over baseline TLS status.

## 3 Association Between TLS and the Efficacy of Neoadjuvant Therapy for NSCLC Through the Immune Mechanisms

### 3.1 Clinical Evidence and the Role of TLS in the Prognosis After Neoadjuvant Therapy

#### 3.1.1 The Correlation Between Pathological Response and Long‐Term Survival

Pathological complete response (pCR) refers to the absence of residual tumor cells detected in the surgically resected tumor tissue after neoadjuvant therapy, while major pathological response (MPR) is usually defined as the proportion of residual tumor cells being lower than a certain threshold [51]. For example, some studies define MPR as tumor cell residue ≤ 10% [51]. pCR and MPR are important indicators for evaluating the efficacy of neoadjuvant therapy and are closely related to the patient's long‐term survival. Clinical studies have shown that lung cancer patients who achieve pCR after neoadjuvant therapy usually have a longer survival period [51]. For example, a study on resectable NSCLC patients who received neoadjuvant chemotherapy combined with immunotherapy showed that the disease‐free survival (DFS) of patients who achieved pCR was significantly better than that of patients who did not achieve pCR [51]. Specifically, the mDFS (median disease‐free survival) of the pCR group was not reached, while the mDFS of the non‐pCR group was 20.24 months (*p* = 0.020) [51]. This indicates that achieving pCR in neoadjuvant therapy can significantly improve the patient's prognosis. The presence of TLS is closely related to the occurrence of pCR/MPR. Studies have found that patients with positive TLS are more likely to achieve pCR or MPR after neoadjuvant therapy [61]. Yang et al.'s [61] study showed that the ratio of TLS to the tumor area can distinguish patients who achieved pCR + MPR from those who did not achieve pCR + MPR among patients who received chemo‐immunotherapy. This indicates that TLS can be used as a potential marker to predict pathological response. Forde et al.'s [62] study also emphasized the potential of TLS as a predictive marker, but the specific pCR/MPR rate data were not given in the provided references. TLS promotes processes such as antigen presentation and T‐cell activation, thereby promoting pathological response. As a place for immune cell aggregation and interaction, TLS can effectively promote the presentation of tumor antigens and activate the anti‐tumor immune response of T cells. B cells mature in TLS and produce specific anti‐tumor antibodies, further enhancing the immune response [63]. These immune responses work together to promote the clearance of tumor cells, thereby achieving pathological remission.

#### 3.1.2 The Predictive Value of TLS Density, Maturity, and Spatial Distribution for Prognosis

The quantitative indicators of TLS include density, maturity, and spatial distribution. TLS density usually refers to the number of TLS per unit area, and maturity is related to the presence of structures such as germinal centers (GC) in TLS [64]. Spatial distribution describes the location of TLS in tumor tissue, such as intra‐tumoral, invasive margin, or stromal area [65]. High‐density and high‐maturity TLS are usually associated with better survival prognosis [64]. Cabrita et al.'s [13] study showed that high‐maturity TLS containing germinal centers was strongly correlated with improved patient survival, but the specific data were not given in the provided references. Zhao et al.'s [64] study also found that as the maturity of TLS increased, the proportions of CD4+ CD103+ TRM and CD8+ CD103+ TRM increased significantly, indicating a better prognosis. Specifically, according to the maturity of TLS, patients were divided into three grades (Grade 1, Grade 2, Grade 3). The proportion of CD103+ TRM in Grade 3 patients (with high‐maturity TLS) was significantly higher than that in grade 1 and Grade 2 patients, and Grade 3 patients with high CD103+ TRM had the best prognosis [64]. Regarding the prognostic significance of TLS spatial distribution, there are controversies in different studies. Some studies have shown that TLS at the tumor edge have more significant prognostic significance [65]. Ding et al.'s [65] study showed that the number of TLS at the tumor boundary was negatively correlated with the number of STAS (tumor spread through air spaces) (*r* = −0.324, *p* < 0.001), and high—density TLS at the tumor boundary were associated with a low‐risk stratification of 14‐gene testing (*p* = 0.013). Multivariate Cox analysis showed that TLS at the tumor boundary were an independent prognostic factor for recurrence‐free survival (HR = 0.856, 95% CI = 0.759–0.966, *p* = 0.026), and mature TLS were an independent prognostic factor for overall survival (HR = 0.841, 95% CI = 0.717–0.988, *p* = 0.035) [65]. This may be because TLS at the tumor edge can more effectively control the invasion and metastasis of tumor cells. However, some studies have also found that intra‐tumoral TLS also have important prognostic value, which suggests that the heterogeneity of the tumor microenvironment may affect the function and prognostic significance of TLS. To better evaluate the prognostic value of TLS, researchers have proposed the concept of a TLS scoring system, which comprehensively evaluates by combining indicators such as density and maturity. For example, the TLS density can be divided into high, medium, and low groups, and the maturity of TLS can be divided into mature and immature categories according to the presence of germinal centers, and then the patient's prognostic risk can be comprehensively evaluated. Xu et al. [66] found that PLR (platelet‐lymphocyte ratio) ≤ 201.8, TLS abundance, and TLS maturity were independent predictors of MPR, and the PLR‐TLS combined model was better than the single‐indicator model in evaluating MPR in NSCLC patients.

### 3.2 Immune Mechanism of Cell Interaction in TLS

As the center of the immune response, the interaction of immune cells inside TLS is crucial for anti‐tumor immunity. These interactions can be divided into three levels: effector, inhibitory, and balanced.

#### 3.2.1 B‐Cell Maturation and the Production of Specific Anti‐Tumor Antibodies

**FIGURE 3:**
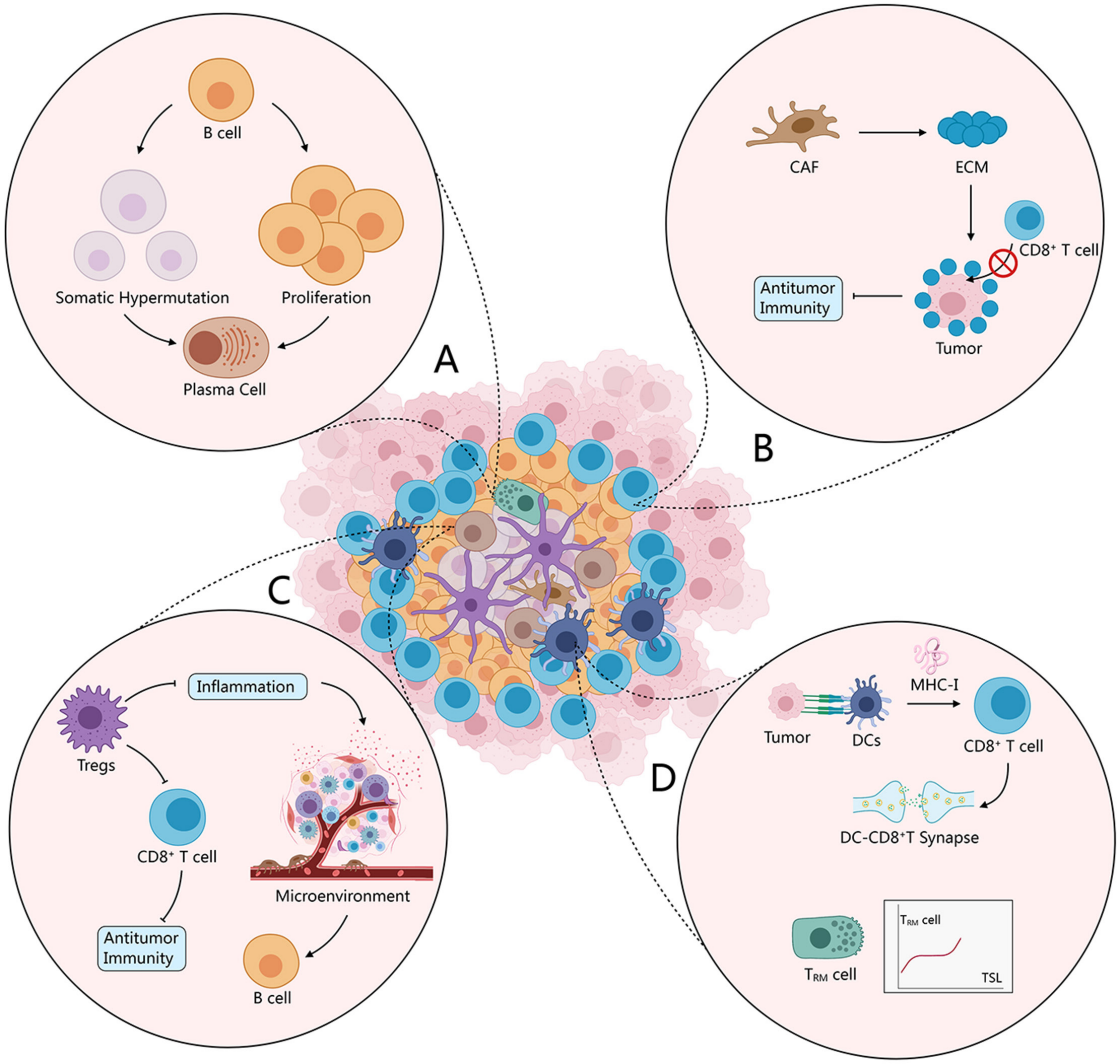
Cellular and molecular mechanisms of TLS‐mediated anti‐tumor immunity. (A) B cell maturation and antibody production. Within TLS, B cells undergo somatic hypermutation, proliferation, and differentiation into plasma cells, which secrete antibodies targeting tumor cells. CAFs and ECM provide structural support and regulate the immune microenvironment. (B) CD8^+^ T cell priming. DCs cross‐present tumor antigens via MHC‐I to CD8^+^ T cells in the presence of inflammatory cues, driving their activation and proliferation. Tregs may modulate this process, contributing to immune homeostasis. (C) DC–CD8^+^ T cell synapse formation. A specialized immunological synapse between DCs and CD8^+^ T cells facilitates sustained TCR signaling, co‐stimulation, and optimal T cell activation, which is critical for effective anti‐tumor immunity. (D) Maintenance of tissue‐resident memory T cells (TRM). TLS support TRM cell maintenance, enabling long‐term, site‐specific immune surveillance and rapid recall responses upon tumor antigen re‐encounter, thereby sustaining durable anti‐tumor immunity. Interplay among B cells, CD8^+^ T cells, and other immune subsets reinforces this protective memory response.

In TLS, B cells undergo maturation and clonal expansion and produce specific anti‐tumor antibodies [63]. The GC is a key site for B‐cell maturation, where B cells undergo clonal expansion and somatic hypermutation (SHM) to improve the affinity of antibodies (Figure 3A) [63]. Studies have shown that tumor‐reactive antibodies (such as anti‐NY‐ESO‐1 antibodies) can directly kill tumor cells or kill tumor cells through antibody‐dependent cell‐mediated cytotoxicity (ADCC). Petitprez et al.'s [63] study found that the B‐cell receptor (BCR) diversity score was related to the treatment response, but the specific data were not given in the provided references. Wu et al.'s [63] study found that CD20+ CD22+ ADAM28+ B cells were present in tumor‐associated TLS and could promote the treatment response of ICI. Analysis of single‐cell transcriptome data from patients who received ICI treatment identified a B‐cell subset [B (IR) (ICI‐Responsive B) cells], which had a memory phenotype. In the TLS of skin squamous cell carcinoma (SCC), renal cell carcinoma (RCC), colorectal cancer (CRC), and breast cancer, the presence of B (IR) cells was confirmed by immunofluorescence [63]. The gene signature related to B (IR) was associated with positive outcomes in melanoma, glioblastoma, NSCLC, head‐and‐neck squamous cell carcinoma (HNSCC), or RCC patients who received ICI treatment, and the density of B (IR) cells predicted the response of NSCLC patients to checkpoint immunotherapy [63]. Consistently, tumor‐bearing mice with B (IR) cell depletion were resistant to ICIs [63].

#### 3.2.2 Activation and Maintenance of CD8+ T Cells in TLS

TLS is an important site for the activation of CD8^+^ T cells and the maintenance of tissue‐resident memory T cells (TRM). Within TLS, dendritic cells (DC) can effectively present tumor antigens to CD8^+^ T cells, forming DC‐CD8^+^ T cell synapses (Figure 3D). Ultrastructural evidence shows the formation of DC‐CD8^+^ T cell synapses within TLS [61]. TRM cells (CD103^+^CD69^+^) are a special type of T cells present in tumor tissues and have local immune surveillance functions. They can reside in the tumor microenvironment for a long time and rapidly activate the immune response when the tumor recurs (Figure 3D) [64]. A study by Zhao et al. [64] found that the higher the maturity of TLS, the higher the proportion of CD103^+^ TRM cells, indicating a better prognosis. Neoadjuvant immunotherapy can induce the expansion of TRM cells, thereby delaying tumor recurrence. Specific data are not given in the references and further research is needed.

#### 3.2.3 Immunosuppressive Components and Their Dual Roles

Although TLS plays an important role in anti‐tumor immunity, there are also immunosuppressive components in it, such as Treg cells and CAFs. The presence of these immunosuppressive components makes the function of TLS more complex.

Treg cells have dual roles in TLS. On the one hand, Treg cells can inhibit excessive inflammation and protect the normal function of the germinal center. On the other hand, Treg cells may also inhibit the activity of effector T cells, thereby weakening the anti‐tumor immune response (Figure 3C). A study by Devi‐Marulkar et al. [67] showed that Treg cells infiltrate tumor‐induced TLS and are associated with poor clinical outcomes in NSCLC patients. Tumor‐infiltrating Treg cells inhibit the proliferation and cytokine secretion of autologous tumor‐infiltrating CD4^+^ T cells. Antibodies against cytotoxic T lymphocyte‐associated protein‐4 (CTLA‐4) and glucocorticoid‐induced tumor necrosis factor receptor‐related protein (GITR) can restore this ability, but other immune checkpoint (ICP) molecules cannot [67]. Treg cells in the entire tumor, including those in TLS, are associated with poor prognosis in NSCLC patients. The combination of TLS DC and CD8^+^ T cells can achieve a higher distinction in overall survival [67]. Therefore, targeting Treg cells (especially in TLS) may represent a major challenge in enhancing the anti‐tumor immune response initiated in TLS [67].

CAF is another important type of cell in the tumor microenvironment. They can secrete extracellular matrix (ECM) components, physically isolate effector cells, and thus inhibit the anti‐tumor immune response (Figure 3B) [68]. Currently, there is a lack of spatial transcriptome data directly citing how the ECM components secreted by CAF physically isolate effector cells, and further research is needed in future studies. A precise strategy for targeting immunosuppression within TLS, such as local Treg depletion combined with immunotherapy, may be an effective treatment method. However, there is currently a lack of relevant clinical data, and further research is required.

### 3.3 Mechanistic Insights From Multimodal Technologies

#### 3.3.1 Cell–Cell Interaction Networks Within TLS

Multimodal technologies have revealed key immune interactions within TLS. Using multicolor immunofluorescence (mIF), co‐localization of B cells and T follicular helper cells has been observed, with close proximity required for germinal center formation [14]. Spatial transcriptomics (e.g., Visium) identifies CXCL13^+^ regions enriched for B‐cell signatures, confirming that chemokine gradients organize TLS architecture [69]. These approaches have also uncovered novel immune checkpoint interactions, though further studies are needed to validate specific candidates.

#### 3.3.2 AI and Radiomics for TLS Functional Inference

Artificial intelligence (AI) applied to radiology images can infer TLS status non‐invasively. Zhao et al. [70] developed a radiomics‐integrated TLS system (RAITS) based on preoperative CT features, which identified TLS in stage I lung adenocarcinoma with an AUC of 0.78 (95% CI: 0.69–0.88) in an external validation cohort. TLS‐positive patients had significantly longer recurrence‐free survival (*p* = 0.02). Such models demonstrate the potential of AI to stratify patients without tissue sampling, though validation in neoadjuvant cohorts is lacking.

## 4 Heterogeneity of TLS and Mechanisms of Treatment Resistance

This chapter systematically explores the balance relationship between the spatial location, maturity of intra‐tumoral TLS, and local immune responses. It also analyzes the influence of tumor genotypes and microenvironmental factors on TLS regulation and the potential role of TLS in immunotherapy resistance. Through multi‐angle analysis, it can be found that there are significant heterogeneities in the composition and function of TLS in different tumor regions and different maturity states. This heterogeneity not only affects the local anti‐tumor immune response but also is closely related to the efficacy after neoadjuvant treatment.

### 4.1 Balance Between TLS Maturity, Spatial Heterogeneity and Local Immune Responses

#### 4.1.1 Functional Differences Between TLS in the Tumor Boundary Area and Intra‐Tumoral TLS

**FIGURE 4:**
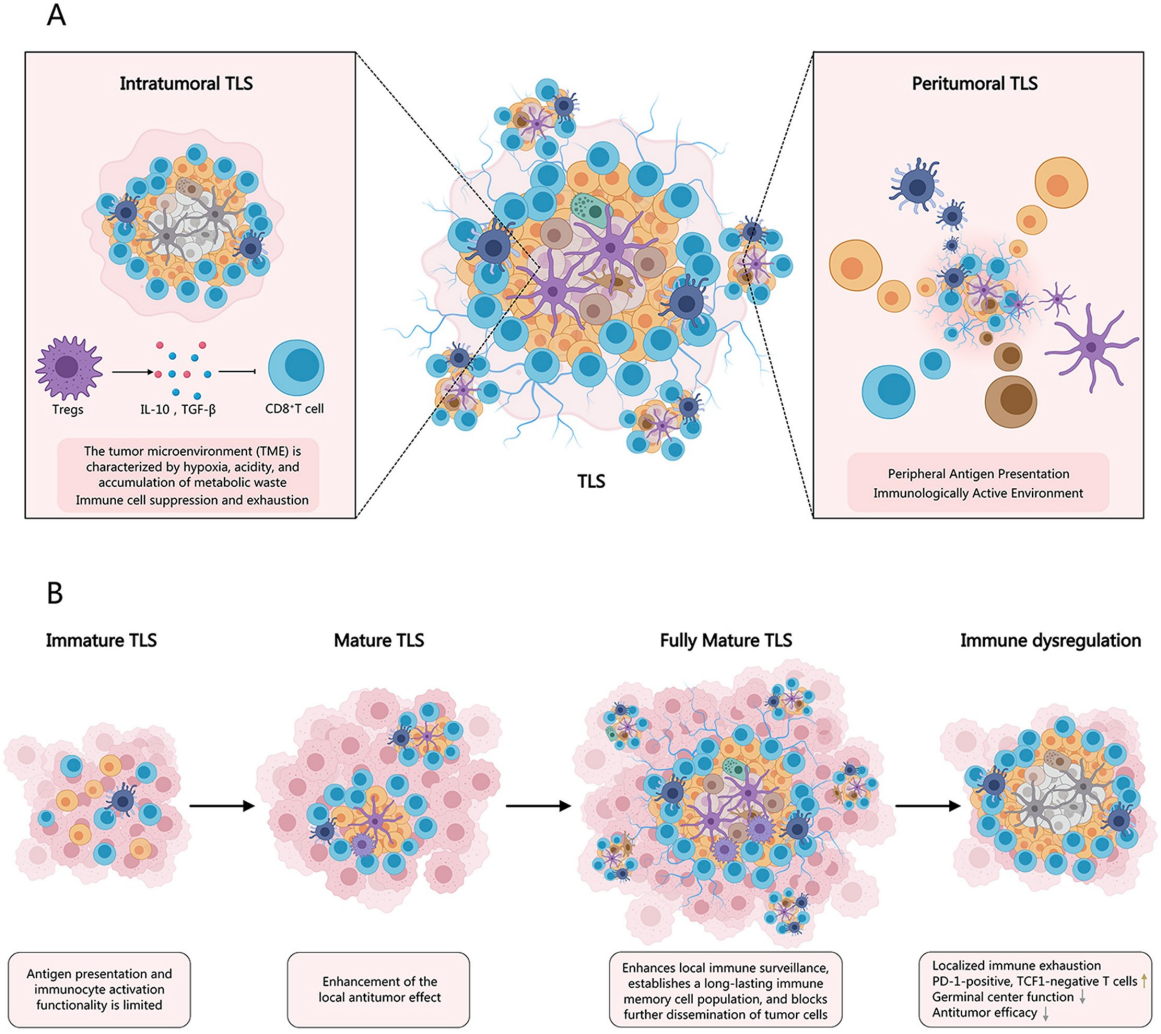
Tertiary lymphoid structures (TLS) in the tumor microenvironment: Localization, maturation, and functional implications. (A) Intratumoral versus peritumoral TLS and their divergent roles in immune modulation. The tumor microenvironment (TME) is characterized by hypoxia, acidity, and metabolic waste accumulation, which drive immune cell suppression and exhaustion. Intratumoral TLS are infiltrated by regulatory T cells (Tregs) that secrete IL‐10 and TGF‐β, impairing CD8^+^ T cell function and promoting tumor immune evasion. Conversely, peritumoral TLS function as immunologically active niches that support peripheral antigen presentation and sustain antitumor immunity. (B) Maturation stages of TLS and associated functional outcomes. TLS maturation progresses through three stages: (i) Immature TLS—characterized by immune dysregulation, localized immune exhaustion with PD‐1^+^TCF‐1^−^ T cell accumulation, and limited functionality; (ii) mature TLS—marked by enhanced local immune surveillance, antigen presentation, immunocyte activation, and initiation of germinal center responses; (iii) fully mature TLS—exhibiting robust germinal center function, memory cell population expansion, and sustained long‐term immunity, which effectively blocks tumor dissemination and correlates with superior antitumor efficacy.

In solid tumors such as lung cancer, the spatial distribution of TLS often shows obvious regional differences, mainly distributed in the tumor boundary area and the intra‐tumoral area. TLS in the tumor boundary area is generally located at the junction of the tumor and the surrounding healthy tissues. These areas can often more effectively recruit peripheral immune cells and form a close synergistic effect with TLS. Due to its relatively open spatial structure and abundant vascular pathways, TLS in the boundary area can often obtain more peripheral antigen‐presenting cells (such as dendritic cells) and B cells, thus forming a relatively active immune activation environment (Figure 4A). For example, in the boundary area, B cells with high expression of chemokines such as CXCL13 are enriched and often show high synergistic anti‐tumor activity with CD8^+^ T cells in the tumor area. The immune response in this area shows strong cytokine secretion and accumulation of effector T cells, which helps to form a local immune microenvironment dominated by antigen presentation and immune activation [61, 71].

In contrast, intra‐tumoral TLS is deeply embedded in the tumor center and is interfered with by unfavorable factors such as hypoxia, acidity, and high accumulation of metabolic products in the tumor microenvironment. The immune cells within it are often in a state of inhibition or exhaustion. Intratumoral TLS may be rich in Treg cells and other immunosuppressive immune cells. These cells limit the activity of effector T cells by secreting inhibitory cytokines such as IL‐10 and TGF‐β, thereby affecting the anti‐tumor immune response [67, 72]. In addition, the formation of intra‐tumoral TLS may also be interfered with by abnormal local tumor metabolism. For example, high‐level glucose uptake and lactic acid accumulation by tumor cells can lead to energy metabolism disorders in local immune cells, indirectly weakening the immune effect (Figure 4A) [72, 73]. Therefore, although TLS also exists in the tumor, its organizational structure has relatively low functionality, and the immune response it activates often cannot fully exert its anti‐tumor effect, which also explains to some extent the poor efficacy of some patients after neoadjuvant treatment.

The latest research on multimodal imaging and spatial transcriptomics technologies shows that in the tumor of the same patient, TLS in the boundary area usually shows higher maturity and activity, with a higher ratio of CD8^+^ T cells to B cells in the cell composition, while intra‐tumoral TLS often shows relatively low maturity and is accompanied by an up‐regulation of immunosuppressive factors. This spatial heterogeneity brings complex regulatory mechanisms to the local immune balance and may also become a target for treatment intervention [65, 74].

#### 4.1.2 Relationship Between TLS Maturity and Anti‐Tumor Immune Effects

There are obvious hierarchical differences in the maturity state of TLS from immature to highly mature. The maturity degree directly determines the quality of the local immune response. Immature TLS mainly presents as simple lymphocyte aggregates, lacking typical germinal centers and HEVs, and has limited antigen‐presenting and immune‐cell activating functions. In contrast, mature TLS has obvious germinal centers and complete neural regulation and vascular tissues, which can support the affinity maturation of B cells, the formation of immune memory, and the continuous activity of effector T cells, thus exerting a more powerful anti‐tumor effect locally (Figure 4B) [64, 75].

After neoadjuvant treatment, clinical studies have found that the presence of mature TLS is significantly correlated with an increase in the degree of pathological remission (such as MPR or even PCR). Such patients often show higher CD8^+^ T cell infiltration and lower immune checkpoint expression, suggesting that mature TLS not only serves as a structural indicator but also reflects the comprehensive effect of local anti‐tumor immunity [51, 76]. In addition, higher‐maturity TLS can gradually enhance local immune surveillance through continuous antigen exposure and cell–cell interactions, forming a persistent population of immune memory cells, thus effectively blocking the re‐spread of tumor cells. This process may be closely related to cell differentiation and antibody affinity maturation in the germinal center and it also reflects the dynamic changes and remodeling of the local immune microenvironment after neoadjuvant treatment [64, 75].

However, it should be noted that an increase in TLS maturity does not always linearly enhance the anti‐tumor effect. When TLS develops to a highly mature state, if the local immune regulation is imbalanced or the continuous antigen stimulation is too strong, it may lead to local immune exhaustion, that is, an increase in PD‐1 positive and TCF1‐negative T cells and a weakening of germinal center function, thus reducing the anti‐tumor effect [75, 77]. Therefore, the precise quantification and dynamic monitoring of TLS maturity will help to better evaluate the prognosis of immunotherapy and provide a reasonable immune regulation strategy for subsequent intervention.

### 4.2 Influence of Tumor Genotypes and Microenvironmental Factors on TLS Regulation

#### 4.2.1 Association Between Driver Gene Status and TLS Formation

In recent years, studies have shown that the driver gene status within the tumor plays a key role in regulating the local immune microenvironment. In particular, the mutation or fusion status of genes such as EGFR and ALK can have a significant impact on the formation of TLS. For example, in some lung cancer patients, EGFR mutations are often accompanied by an up‐regulation of angiogenesis factors such as VEGF, which may inhibit the infiltration of local lymphocytes, resulting in a decrease in the density and insufficient maturity of TLS [77, 78]. In EGFR‐mutated tumors, low‐density TLS and a weak anti‐tumor immune response are often associated with a poor prognosis, suggesting that the driver gene status may serve as a potential screening marker for TLS induction strategies.

On the contrary, although the ALK fusion state shows an immunosuppressive state in some studies, in some models, it has also been observed that the activation of the JAK–STAT pathway promotes the secretion of chemokines such as CXCL13, thereby enhancing the formation of TLS [77, 78]. In general, different driver gene states indirectly affect the recruitment of local immune cells and the formation of TLS by regulating the cytokines, chemokines, and antigen‐presenting molecules secreted by tumor cells. Clinically, the tumor genotype not only indicates the activation status of intracellular signaling pathways but also largely reflects the construction status of the tumor immune microenvironment and the functional performance of TLS, which provides a theoretical basis for precise targeting and combined immunotherapy [72, 73].

#### 4.2.2 Local Immunosuppressive Network and Competition Relationship

The complex immunosuppressive network in the local tumor immune microenvironment plays an important regulatory role in the formation and function of TLS. Within and around TLS, there are various immunosuppressive cell populations, such as Tregs, myeloid‐derived suppressor cells (MDSCs), tumor‐associated macrophages (TAMs). Tregs often exert immunosuppressive effects in the form of molecules such as CTLA‐4 and LAG‐3 within TLS, thereby affecting the activity of effector T cells and the antigen‐presenting function within TLS [67, 72].

In addition, collagen deposition and other matrix components secreted by CAF in the local extracellular matrix may also have a physical isolation effect on the formation and function of TLS, limiting the cell–cell contact between effector cells and antigen‐presenting cells [63, 79]. This competition relationship is not only reflected in the competition for local resources and living environment among various immune cells but also in the local regulation of cytokine and chemokine concentrations. For example, in some studies, it has been found that a high local concentration of IL‐10 and TGF‐β can inhibit the expression of the chemokine CXCL13, thereby reducing the recruitment of B cells and T cells to TLS. This phenomenon is particularly obvious inside the tumor, thus exacerbating regional immune tolerance and drug resistance [66, 79]. In addition, single‐cell sequencing technology also shows that in some tumors, there is an obvious functional confrontation between immunosuppressive immune cells and activated immune cells. This confrontation state determines whether TLS can maintain a long‐term anti‐tumor effect in the local immune microenvironment [65, 80]. Overall, the existence of the local immunosuppressive network is closely related to the regulation of various cytokines and chemokines. Its complex cell competition relationships and signal transduction networks significantly affect the formation and maturation of TLS. In the future, intervening in this negative regulatory mechanism, including blocking Tregs, MDSCs, or regulating the function of CAFs, may become an important strategy to promote TLS formation, reshape the immune microenvironment, and improve the efficacy of immunotherapy.

### 4.3 Potential Role of TLS in Immunotherapy Resistance

#### 4.3.1 Accumulation of Immunosuppressive Components in TLS and Its Impact on Antitumor Immunity

Although TLS is generally considered to be closely associated with better prognosis and immunotherapy response, in some cases, the accumulation of immunosuppressive components within TLS may lead to a local immunosuppressive state, thereby affecting the overall efficacy of immunotherapy. For example, in NSCLC patients, the accumulation of Tregs and IL‐10^+^ Bregs in TLS restricts the function of locally increased CD8^+^ T cells, reducing the efficacy of anti‐PD‐1/PD‐L1 antibody therapy [67, 72]. Moreover, studies have shown that under long‐term immunotherapy pressure, immune exhaustion may occur in TLS, typically manifested by the aggregation of PD‐1 positive and TCF1‐negative T cells and the gradual atrophy of the germinal center structure, ultimately leading to a weakened local immune effect [75, 77]. These data indicate that the accumulation of immunosuppressive components in TLS has a dual effect: on the one hand, moderate suppression helps regulate immune balance, but excessive suppression may disrupt the continuity of local antitumor immunity and become an important internal factor for immunotherapy resistance. In clinical practice, evaluating the immune cell lineages and distribution of inhibitory factors in TLS through pathology and multicolor immunofluorescence techniques helps reveal the characteristics of the immune microenvironment in patients with immune resistance, providing a basis for subsequent combined immune regulation interventions. For cases with a high proportion of Tregs or inhibitory B cells in TLS, using combined CTLA‐4 or other immune checkpoint inhibitors may restore local antitumor immunity to some extent and improve the treatment effect [81, 82].

#### 4.3.2 Imbalance of TLS Regulation and the Local Immune Exhaustion Models

Based on a comprehensive analysis of the composition and functional status of immune cells in TLS, scholars have proposed the “TLS exhaustion” hypothesis. That is, during continuous antigen stimulation and immune activation, effector cells in TLS gradually lose their activity, accompanied by the up‐regulation of inhibitory molecules such as PD‐1 and the failure of germinal center function, ultimately forming a local immune‐exhausted microenvironment. This model not only explains the limited efficacy of immune checkpoint inhibitors in some patients but also provides an important idea for reversing drug resistance. For example, through combined means such as chemokine regulation, immune metabolism intervention, and low‐dose radiotherapy (LDRT), it is expected to reshape the internal structure and function of TLS, thereby restoring local immune activity and improving the response rate of immunotherapy [74, 81]. Currently, in some prospective clinical trials, researchers have attempted to incorporate TLS‐related indicators into the stratified analysis of immunotherapy and explore strategies to improve efficacy by intervening in the structure and cell function of TLS. For example, the combined application of CCL19‐mediated cytokine therapy and PD‐1 inhibitors shows the potential to reverse immune exhaustion in TLS and enhance the function of CD8^+^ T cells in a certain patient population [81, 82, 83, 84].

In addition, in the dynamic tracking of tumors, using spatial transcriptomics and single‐cell sequencing technologies can not only monitor the changes in TLS maturity in real time but also track the evolution of key immune cell clones, providing intuitive evidence for understanding immune resistance.

Generally, the model of TLS regulation imbalance and local immune exhaustion reveals a complex and dynamic local immune network. In the early stage of immunotherapy, TLS plays a positive regulatory role by promoting antigen presentation and effector cell activation. However, under long‐term stimulation, immunosuppressive components gradually accumulate in the local microenvironment, leading to immune exhaustion and affecting the treatment effect. In response to this mechanism, future research should focus on exploring how to balance the ratio of pro‐effector and inhibitory cells in TLS through precise immune intervention strategies, restore local immune vitality, overcome immune resistance, and provide a basis for formulating individualized treatment plans for patients.

Collectively, TLS plays an important positive regulatory role in antitumor immunity in the local immune response after neoadjuvant therapy for NSCLC. However, it may also lead to drug resistance under specific conditions due to internal immune regulation imbalance. Through in‐depth analysis of the functional differences of TLS in different spatial regions, maturity stratification, and the immunosuppressive network inside and outside the tumor, we can more comprehensively understand the complexity of the lung cancer immune microenvironment and provide theoretical support and practical guidance for combined immunotherapy strategies based on TLS regulation.

## 5 Exploration of TLS as a Biomarker and Therapeutic Target

### 5.1 Technical Considerations for TLS Detection in Post‐Neoadjuvant Specimens

The application of TLS detection methods to samples from patients who have received neoadjuvant chemoimmunotherapy presents unique challenges not encountered in treatment‐naïve tissues. Post‐treatment specimens often contain small residual tumor foci, extensive necrosis, fibrosis, and regression beds, which can fragment TLS structures or obscure their identification [85]. Moreover, baseline (pre‐treatment) biopsies are typically small core needle samples, limiting the assessment of TLS spatial organization [86]. Therefore, the choice of detection technique must balance sensitivity, resolution, and compatibility with degraded or limited tissue.

#### 5.1.1 H&E and Immunohistochemistry (IHC): Utility and Limitations

H&E is first‐line but suffers from subjective maturity grading, especially when germinal centers are disrupted by therapy. IHC (CD20, CD3, CD21, PD‐1) improves cell‐subset identification but faces inconsistent antigen retrieval in post‐treatment tissues and is operator‐dependent [12]. Digital pathology (QuPath, HALO) improves reproducibility, but necrosis/fibrosis must be manually excluded, introducing bias.

#### 5.1.2 Multicolor Immunofluorescence (mIF)

mIF enables simultaneous detection of multiple markers (e.g., CD20, CD3, CD21, PD‐1, FoxP3) while preserving spatial relationships [87]. In post‐neoadjuvant samples, mIF distinguishes therapy‐induced lymphoid aggregates (often lacking mature follicular dendritic cell networks) from true functional TLS [88]. However, mIF requires high‐quality FFPE or fresh‐frozen tissue [89]; neoadjuvant samples frequently exhibit autofluorescence from necrotic debris, and antibody panels may fail in fibrotic areas.

#### 5.1.3 Spatial Transcriptomics—Practical Constraints

Spatial transcriptomics (10× Visium, MERFISH, CODEX) provides “structure–function‐molecule” integration and has revealed TLS‐adjacent upregulation of CXCL13 and immune checkpoints in neoadjuvant lung cancer [90]. However, high cost and intact tissue requirements limit its use; small post‐treatment biopsies (≤ 5 mm) often yield insufficient capture area [91].

#### 5.1.4 Deep Learning (DL) Models for TLS Quantification

Convolutional neural networks (CNNs) can automatically identify TLS on H&E or IHC slides. Their main advantage for neoadjuvant samples is the ability to learn therapy‐altered morphologies (e.g., regressed germinal centers) that humans may miss [92]. However, DL requires large, well‐annotated training datasets, which are scarce for post‐treatment specimens [93]. Transfer learning from treatment‐naïve cohorts performs poorly because TLS histology changes after therapy [12].

#### 5.1.5 Recommended Tiered Approach for Post‐Treatment Analysis

**FIGURE 5:**
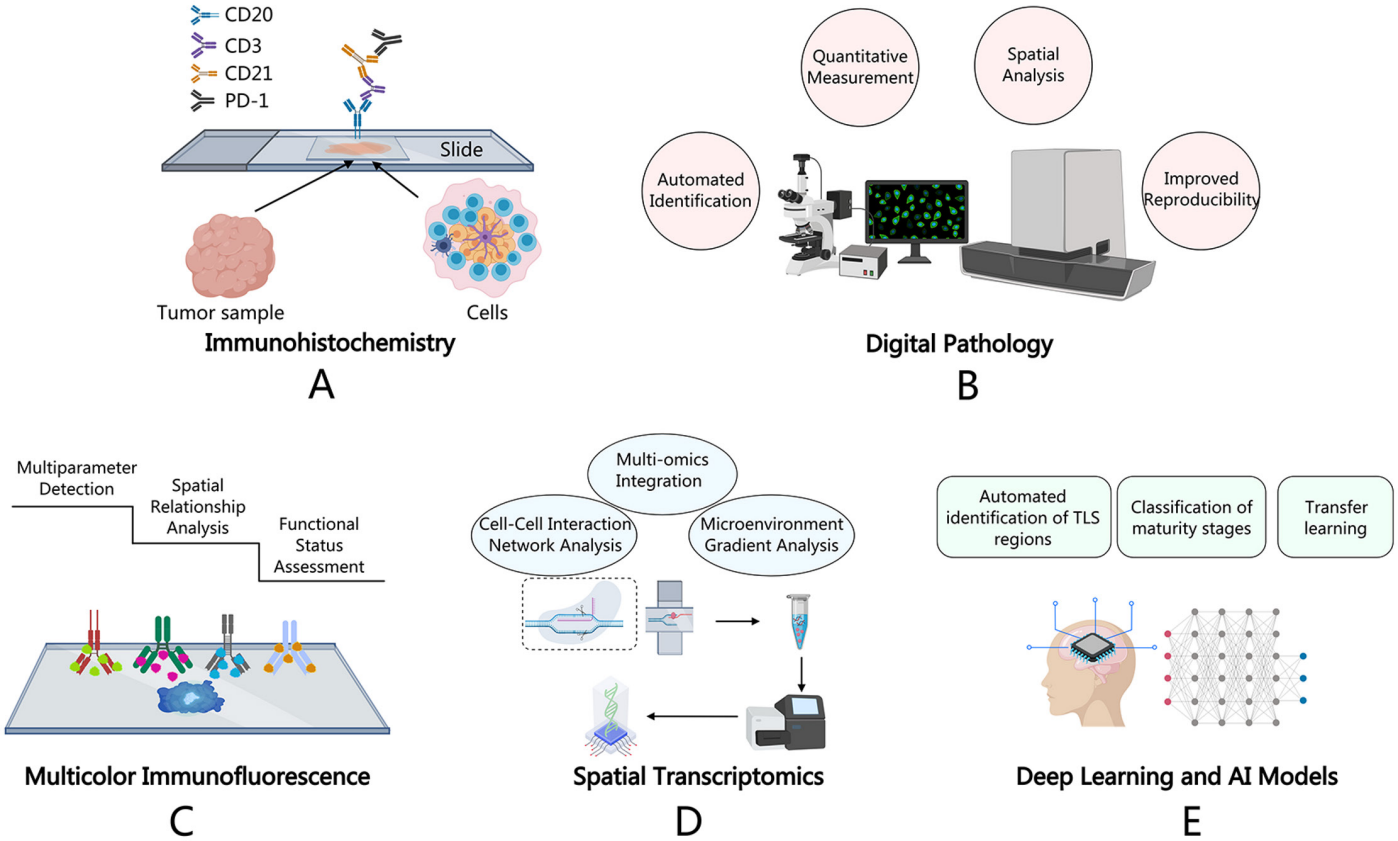
Methodological approaches for TLS detection, characterization, and clinical translation. (A) Tumor sample processing and immunohistochemistry. Tumor tissue sections are stained with antibodies against CD20 (B cells), CD3 (T cells), CD21 (follicular dendritic cells), and PD‐1 to enable spatial localization of immune populations. (B) Multicolor immunofluorescence (mIF). mIF enables simultaneous detection of multiple markers on a single section, facilitating multiparameter detection, spatial relationship analysis, and functional status assessment of immune cells within TLS. (C) Digital pathology and spatial analysis. High‐resolution imaging combined with digital tools enables quantitative measurement, automatic identification, and classification of TLS regions. Multi‐omics integration, cell–cell interaction network analysis, and microenvironment gradient analysis provide deeper insights into TLS architecture and function. (D) Deep learning and artificial intelligence models. AI‐powered algorithms, particularly deep learning architectures with transfer learning capabilities, enable automatic TLS identification, classification, and quantification in large‐scale histopathological datasets, enhancing reproducibility and throughput. (E) Integrated analytical pipeline. The convergence of high‐plex imaging, digital pathology, spatial transcriptomics, and AI‐based classification creates a comprehensive framework for automated TLS detection, functional characterization, and clinical outcome correlation, facilitating the translation of TLS‐based biomarkers into routine clinical practice.

Tier 1 (clinical routine): H&E + IHC (CD20, CD3, CD21) with digital pathology. Explicitly exclude necrotic/fibrotic areas (Figure 5A,B).

Tier 2 (research/prospective trials): mIF on FFPE sections (≤ 4 markers) to assess TLS maturity and CD8^+^/Treg ratio (Figure 5C).

Tier 3 (exploratory): Spatial transcriptomics only when ample tissue (≥ 10 mm^2^ viable tumor bed) is available. Deep learning requires institution‐specific retraining (Figure 5D,E).

The major limitation is lack of standardization. Future studies should report pre‐analytical parameters (biopsy size, necrotic fraction, fixation time) and use paired pre−/post‐treatment samples whenever possible.

### 5.2 Integrating TLS Scoring Systems With Multi‐Omics: Focus on Neoadjuvant Cohorts

Traditional TLS scoring (maturity, density, spatial distribution) needs modification for neoadjuvant specimens because therapy can artificially increase or decrease TLS counts due to tumor shrinkage and immune cell infiltration into regression beds [23].

#### 5.2.1 Quantitative Indicators Adapted for Post‐Treatment Samples

##### 5.2.1.1 Maturity Grading

The conventional three‐tier system (immature aggregates, primary follicles, mature TLS with germinal centers) is often ambiguous in treated tissues. A more robust approach uses CD21^+^ follicular dendritic cell networks as a binary indicator of maturity (present vs. absent), which is less affected by therapy [94]. Alternatively, AID (activation‐induced cytidine deaminase) expression by in situ hybridization can confirm ongoing germinal center reactions [95].

##### 5.2.1.2 Density Calculation

TLS density (number per mm^2^ or percentage of tumor area) must be normalized to viable tumor‐containing tissue only, excluding necrosis and dense fibrosis. Digital pathology software can automate area selection, but manual curation is still required. In small core biopsies (≤ 3 mm), density estimates are unreliable; reporting TLS presence/absence is more honest.

##### 5.2.1.3 Spatial Distribution

In neoadjuvant specimens, TLS located within regression beds (areas of tumor replacement by immune infiltrate and fibrosis) may have different prognostic value than those in residual tumor [62]. Geographic information system (GIS) methods (e.g., distance to nearest residual tumor cell) can be applied, but which require whole‐slide imaging.

#### 5.2.2 Multi‐Omics Integration for Neoadjuvant Response Prediction

Integrating TLS pathological scores with transcriptomic and immunomic data can overcome some limitations of morphology alone. Transcriptomic TLS signatures (e.g., CXCL13, ICOS, B‐cell signature) have been validated in neoadjuvant cohorts [96, 97, 98]. For example, Zhou et al. [99] constructed a four‐gene TLS score (CSF2, CXCL13, IL1R2, and SGPP2) that predicted prognosis and immune‐related features in ccRCC.

##### 5.2.2.1 Important Caveat for Neoadjuvant Samples

Because tertiary lymphoid structures (TLS) comprise multiple immune and stromal cell populations [7], and because neoadjuvant therapy induces widespread transcriptional reprogramming in abundant non‐TLS cell types—most notably fibroblasts and macrophages—the interpretation of bulk transcriptomic data from post‐treatment specimens is inherently confounded. Tissue‐level measurements cannot resolve TLS‐specific transcriptional changes from the therapy‐driven signals originating in surrounding stromal compartments [100]. Thus, spatial transcriptomics or laser‐capture microdissection of TLS regions is preferred, though technically challenging in small samples.

Immunomic data (e.g., flow cytometry of peripheral blood, Olink proteomics) can complement tissue‐based scores. However, whether peripheral blood B‐cell activation mirrors intratumoral TLS after neoadjuvant therapy remains unproven [101].

Machine learning models combining pathological TLS scores, gene expression, and clinical variables have been developed, but external validation in independent neoadjuvant cohorts is lacking. Overfitting is a major risk due to small sample sizes typical of neoadjuvant trials [102].

##### 5.2.2.2 Practical Recommendation

For clinical implementation in the neoadjuvant setting, a simple combined score (e.g., TLS density on H&E + CD8^+^ IHC) is more feasible than multi‐omics. High‐throughput techniques should remain exploratory until standardized protocols for post‐treatment tissue are established.

### 5.3 Personalized Treatment Strategies and Combination Interventions Based on TLS Regulation

#### 5.3.1 Strategies for Directly Inducing TLS Maturity

Since TLS is related to the immune response and prognosis of tumors, can we actively regulate TLS through artificial intervention to enhance the anti‐tumor immune response and optimize neoadjuvant therapy?

##### 5.3.1.1 Chemokine Intervention

The CXCL13/CXCR5 axis plays a central role in B cell recruitment and TLS initiation [81]. CXCL13 is a chemokine that can specifically bind to the CXCR5 receptor and guide B cells to migrate to the TLS area. Therefore, chemokine intervention can be carried out through the following methods [103, 104].

##### 5.3.1.2 Recombinant Protein

Inject recombinant CXCL13 protein into the tumor tissue to directly recruit B cells to the TLS area [105].

##### 5.3.1.3 Gene Vector

Use a gene vector to transfect the CXCL13 gene into tumor cells or immune cells, enabling these cells to express CXCL13 and recruit B cells to the TLS area [106].

##### 5.3.1.4 mRNA Therapy

Use mRNA therapy to deliver CXCL13 mRNA to the tumor tissue, allowing tumor cells or immune cells to translate the CXCL13 protein and recruit B cells to the TLS area [107].

In addition, other factors (such as LTα, CCL19/21) are also involved in the formation and maintenance of TLS and can be considered for combined use with other chemokines to enhance the induction effect of TLS [108].

Immunomodulatory Effects of Low‐Dose Radiotherapy (LDRT): LDRT can activate the STING pathway through DNA damage signals, promote DC activation and chemokine release, and indirectly induce TLS formation [74]. The advantages of LDRT are as follows:

##### 5.3.1.5 Non‐Invasiveness

LDRT is a non‐invasive treatment method with less damage to patients [109].

##### 5.3.1.6 Systemic Effect

LDRT cannot only act on the local tumor but also produce a systemic effect by activating the immune system [110].

##### 5.3.1.7 Synergistic Effect

LDRT can synergize with other treatment methods such as immunotherapy [111] and targeted therapy [112] to improve the treatment effect.

##### 5.3.1.8 Other Potential Means

In addition to chemokines and LDRT, there are other potential means to promote TLS formation, such as TLR agonists, STING agonists, and oncolytic viruses. TLR agonists can activate the innate immune system, promote dendritic cell activation, and induce the release of chemokines critical for immune cell recruitment [113]. Local administration of TLR agonists has been shown to remodel the tumor immune microenvironment and induce organized intratumoral immune infiltrates [114], suggesting their potential to facilitate tertiary lymphoid structure formation. STING agonists can activate the STING pathway, promote the production of type I interferons, and enhance the anti‐tumor immune response [115]. Oncolytic viruses can selectively infect and kill tumor cells, release tumor antigens and immune‐stimulating factors, and induce TLS formation [116].

Inducing TLS in the neoadjuvant stage has more advantages because the tumor burden is still controllable at this time, and the immune system is not severely exhausted [117]. In addition, neoadjuvant therapy can also eliminate micro‐metastatic foci and reduce the risk of post‐operative recurrence [118]. Therefore, inducing TLS in the neoadjuvant stage can create better conditions for subsequent treatment and improve the survival rate of patients.

#### 5.3.2 Personalized Combination Strategies Guided by TLS‐Based Multi‐Omics Models

##### 5.3.2.1 Synergistic Mechanisms With Immune Checkpoint Inhibitors (ICIs)

ICIs (e.g., PD‐1/PD‐L1 inhibitors) relieve T‐cell exhaustion within TLS and maintain effector function [119]. The hypothesis that high TLS abundance predicts ICI response has been supported: patients with high TLS density show higher response rates and longer survival [75].

##### 5.3.2.2 Metabolic Regulation

Acidosis, hypoxia, and nutrient competition in the tumor microenvironment inhibit TLS maintenance. Targeting the IDO pathway [120], adenosine axis [121], or lactate transporters [7, 122] can improve the immune metabolic environment and support TLS function.

Example of personalized combination design based on TLS status. (i) Tumors lacking TLS: First induce TLS with LDRT or CXCL13 agonists, then initiate ICI. (ii) Tumors with existing but functionally suppressed TLS: Combine ICI with metabolic regulators and Treg‐targeted therapy. (iii) Multi‐omics and AI integration for personalized design: To move beyond static TLS assessment, we integrate multi‐omics data (genomics, transcriptomics, proteomics, spatial omics) and AI models to predict individual responses. For example, Chao et al. [123] used multi‐omics to elucidate cellular origins and TME dynamic evolution in NSCLC and Zhou et al. [99] constructed a four‐gene TLS score (CSF2, CXCL13, IL1R2, and SGPP2) that predicts prognosis and immune‐related features in ccRCC. Machine learning (LASSO, random forests, neural networks) can combine TLS pathological scores (density, maturity, spatial distribution) with gene expression and clinical variables. Challenges include data standardization (ComBat correction) and model interpretability (Shapley values, LIME). Future directions include using spatial transcriptomics and mIF with AI to analyze intratumoral TLS heterogeneity.

### 5.4 Integrated Multi‐Technology Framework for TLS

The integration of the technologies outlined in Table 2 reflects a pivotal shift in TLS assessment: from qualitative annotation to quantitative, spatially resolved, and clinically accessible biomarker profiling. This evolution is essential for leveraging TLS as a predictive and prognostic tool, particularly as neoadjuvant immunotherapy and combination regimens become standard of care across multiple tumor types.

1. Foundational pathology and digital quantification. H&E with immunohistochemistry (IHC) remains the clinical cornerstone for baseline TLS identification and maturity grading, given its low cost and broad availability with FFPE tissues [53, 160]. Expert pathology on H&E is the current reference standard for TLS screening but lacks sensitivity in detecting TLS maturation, and its limitations in inter‐observer reproducibility are well recognized, with interrater agreement rates for TLS presence reported at κ = 0.65 (95% CI, 0.46–0.90) [132]. Digital pathology platforms like QuPath and HALO address this by enabling automated, observer‐independent quantification of TLS density and area, providing a scalable solution for standardized clinical reporting [125, 128]. For instance, studies in metastatic colorectal cancer have shown that median TLS counts increased progressively from H&E to visual mIHC and digital mIHC, reflecting the higher sensitivity of digital approaches [125]. In soft tissue sarcoma, the HALO platform has been used to digitally score multiplex IHC‐stained tissue microarrays for TLS‐associated immune cells, while QuPath has been applied for single‐plex IHC scoring, demonstrating the feasibility of automated digital workflows for TLS assessment [128].
2. High‐dimensional spatial profiling. Multiplex immunofluorescence (mIF) extends beyond routine IHC by preserving spatial context while enabling the simultaneous assessment of functional T‐cell subsets [134, 161]. This is clinically relevant, as studies demonstrate that the balance between CD8^+^ effector T cells and FOXP3^+^ regulatory T cells (Tregs) within TLS significantly influences patient outcomes, with a high CD8^+^/Treg ratio correlating with improved survival [134, 162]. Spatial transcriptomics, such as the 10× Visium platform, adds another layer by revealing chemokine gradients and identifying specific cellular niches, such as CXCL13^+^ B‐cell zones, that are critical for functional TLS activity and correlated with immunotherapy response [140, 141, 163].
3. Genomic resolution and clonal tracking. Single‐cell RNA sequencing combined with T‐cell receptor (TCR) and B‐cell receptor (BCR) tracking provides the highest resolution, identifying treatment‐responsive clonal expansions. This clonal perspective is crucial; for instance, CXCL13^+^ T cells and clonally expanded B cells within TLS are strong correlates of pathologic complete response (pCR) to neoadjuvant therapy, highlighting the active role of TLS in orchestrating adaptive anti‐tumor immunity. In hepatocellular carcinoma treated with neoadjuvant immune checkpoint blockade, high intratumoral TLS density was associated with expansion of the intratumoral T and B cell repertoire and improved relapse‐free survival [150]. Similarly, in nasopharyngeal carcinoma, single‐cell and spatial transcriptomic analyses identified CXCL13^+^ CD8^+^ T cells and germinal center B cells within TLS as key populations linked to immunotherapy response and tumor cell apoptosis [146]. In non‐small cell lung cancer, patients achieving pCR following neoadjuvant chemoimmunotherapy exhibited a more clonal baseline BCR repertoire that was better conserved and reinvigorated during treatment, with BCR metrics emerging as promising predictive biomarkers [12]. In esophageal squamous cell carcinoma, single‐cell and spatial transcriptomic analyses revealed that CXCL13^+^CD103^+^ exhausted T cells within TLS‐associated cellular neighborhoods were significantly enriched in pCR patients and linked to mature TLS formation [164]. Furthermore, in cervical cancer, single‐cell RNA and TCR sequencing identified tumor‐specific germinal center B cells associated with TLS and favorable clinical outcomes [165].
4. Non‐invasive prediction and artificial intelligence. The most transformative advances are in non‐invasive prediction. AI radiomics models, such as the rTLS predictive model developed for triple‐negative breast cancer, demonstrate that routine CT imaging can predict TLS status and treatment response to neoadjuvant therapy, with robust predictive performance across various patient subgroups [152]. In hepatocellular carcinoma, a combined machine learning model integrating preoperative CT imaging and clinical data achieved high predictive accuracy for intratumoral TLS expression, with AUCs of 0.947 in the training set and 0.909 in the validation set [153]. Another multicenter study in HCC further validated a combined model for TLS prediction, achieving AUCs of 0.830–0.831 in validation and test cohorts, with predicted TLS‐positive status correlating with longer progression‐free and overall survival [154]. In stage I lung adenocarcinoma, the Radiomics Integrated TLSs System (RAITS), a multimodal predictive model based on preoperative CT radiomic features, demonstrated superior performance in identifying TLSs, with TLS‐positive patients showing significantly higher recurrence‐free survival [70]. Concurrently, deep learning on histology slides has shown robust performance in predicting pathological response to neoadjuvant immunotherapy. A multi‐instance learning model (GLAM) applied to H&E‐stained whole‐slide images in lung cancer achieved AUCs of 0.951 for predicting major pathological response and 0.864 for predicting pathological complete response [156]. Pathology foundation models have also emerged as powerful tools for automated TLS detection directly from H&E images, with models such as PLIP and CTransPath demonstrating strong performance in identifying TLS within pancreatic ductal adenocarcinoma samples (AUC = 0.94 and 0.89, respectively) [124]. A spatial multi‐omics study in pancreatic cancer further showed that machine learning‐enabled H&E image classification models could characterize TLS‐associated cellular states, revealing a TLS‐specific spatial gene expression signature significantly associated with improved survival. These approaches are particularly valuable because conventional TLS identification requires resource‐intensive techniques that limit routine clinical guidance [124, 152].

**TABLE 2:** Key technologies for TLS assessment—Advantages and clinical applications.

Technology	Key advantages	Clinical applications in TLS assessment
H&E + IHC [53, 124, 125, 126, 127]	Low cost, widely available, compatible with FFPE	Baseline TLS identification; maturity grading (CD20, CD3, CD21) in routine pathology
Digital pathology (QuPath, HALO) [128, 129, 130, 131, 132, 133]	Automated quantification, reduces observer variability	TLS density (number/mm^2^) and area percentage measurement
Multicolor immunofluorescence (mIF) [134, 135, 136, 137, 138, 139]	Multiplexed detection (≥ 4 markers), preserves spatial context	Distinguishes therapyinduced aggregates from functional TLS; assesses CD8+/Treg ratio—
Spatial transcriptomics (Visium, MERFISH) [140, 141, 142, 143, 144, 145, 146]	Gene expression with spatial resolution; reveals chemokine gradients	Identifies CXCL13^+^ B‐cell niches; maps TLS‐tumor interface interactions
Single‐cell RNA‐seq + TCR/BCR tracking [147, 148, 149, 150, 151]	Clonal resolution; tracks immune cell evolution	Identifies treatment‐responsive T/B cell clones; correlates clonal expansion with pCR
AI radiomics (e.g., RAITS) [70, 152, 153, 154, 155]	Non‐invasive; uses routine CT images	Predicts TLS status and recurrence‐free survival without biopsy
Deep learning on histology [102, 124, 132, 156, 157, 158, 159]	Learns therapy‐altered morphologies	Automates TLS detection in post‐neoadjuvant specimens (requires retraining)

Thus, the consolidated framework integrates TLS induction/modulation strategies with multi‐omics‐driven personalization, directly linking biomarker assessment to treatment selection.

## 6 Future Frontiers and Challenges

### 6.1 Novel Technologies for TLS Dynamic Tracking and Immune Cell Clonal Evolution

#### 6.1.1 Single‐Cell Sequencing and TCR/BCR Clonal Tracking

The advent of single‐cell RNA sequencing (scRNA‐seq) and high‐throughput TCR/BCR repertoire reconstruction technologies has enabled precise dissection of the transcriptomic landscapes and clonal evolutionary trajectories of immune cells residing within TLS at single‐cell resolution. scRNA‐seq not only yields high‐dimensional gene expression profiles delineating cell types and states [166, 167], but also, when coupled with TCR/BCR reconstruction algorithms, permits tracking of clonal expansion and migration across distinct spatial compartments and temporal phases [168].

##### 6.1.1.1 Principle of Single‐Cell Transcriptomic Sequencing

Single‐cell suspensions obtained via ultrasonic or enzymatic tissue dissociation are subjected to high‐throughput sequencing utilizing microfluidic chip‐ or microbead‐based capture systems. Unique cell barcodes and unique molecular identifiers (UMIs) ensure independent transcriptome measurement for each individual cell while mitigating batch effects [169].

##### 6.1.1.2 TCR/BCR Clonal Tracking Methodology

V(D)J region sequences of TCRα/β or BCR heavy and light chains are simultaneously captured from scRNA‐seq data and reconstructed using specialized algorithms [170] (e.g., TraCeR [171], VDJtools [172]). Integration of clonal TCR/BCR sequences with transcriptional states facilitates inference of the differentiation status, surface marker expression profiles, and functional zonation of specific clones within TLS [173]. Cross‐timepoint or cross‐regional comparison of clonal composition enables longitudinal monitoring of tumor antigen‐specific T and B cell clonal dynamics.

##### 6.1.1.3 Potential Applications in Neoadjuvant Therapy for Lung Cancer

Single‐cell TCR/BCR tracking holds the potential to identify high‐frequency clonal populations associated with pCR or MPR, which may serve as candidate predictive biomarkers in future studies [12, 174]. Clonal diversity indices (e.g., Shannon index) and clonal expansion metrics can inform individualized neoadjuvant regimen design [175, 176]. Integration with tumor spatial omics further reveals the localization and migratory trajectories of anti‐tumor clones across TLS maturation states and spatial distribution zones, thereby providing a mechanistic basis for the precise modulation of TLS architecture [177, 178].

#### 6.1.2 Application of High‐Resolution Spatiotemporal Imaging in Dynamic Monitoring

Tumor‐associated TLS represent highly dynamic immune cell communities; the multicellular interactions and tissue remodeling processes involving diverse immune subsets necessitate high‐resolution spatiotemporal imaging modalities for real‐time observation and quantification [179, 180]. Recent advances in multiphoton microscopy, light‐sheet microscopy, and in vivo imaging have progressively matured, offering new avenues for dynamic TLS monitoring [179, 181, 182].

##### 6.1.2.1 Light‐Sheet Imaging and Three‐Dimensional Reconstruction

Light‐sheet microscopy facilitates rapid volumetric imaging of whole tumor specimens, generating high‐resolution spatial distribution maps of immune cell populations [184, 185]. Co‐registration with computed tomography enables longitudinal comparison of TLS volume, morphology, and the proportional composition of internal cellular subsets across treatment time points [179].

##### 6.1.2.2 In Vivo Imaging and Molecular Reporters

Systemically administered fluorescent or biotin‐labeled tumor antigens or immune reporter molecules (e.g., Ca^2+^ indicators) enable real‐time capture of molecular signals upon immune cell activation at the organismal level [180]. Comparative analysis across distinct neoadjuvant treatment groups permits longitudinal evaluation of TLS activity and intracellular calcium dynamics, thereby offering a potential platform for rapid therapeutic efficacy assessment [179, 183].

##### 6.1.2.3 Research Significance

Spatiotemporal multimodal imaging enables direct visualization of the structural and functional remodeling trajectories of TLS pre‐ and post‐treatment, facilitating the discovery of actionable regulatory nodes [179]. Furthermore, it provides a mechanistic basis for intervention strategy design, including the determination of optimal temporal windows for low‐dose radiotherapy or chemotherapy‐induced TLS formation and the timing of combinatorial immune checkpoint inhibitor administration [184].

### 6.2 Interdisciplinary Integration and Mechanistic Advances

#### 6.2.1 Multi‐Omics Data Integration and AI Model Construction

Elucidating TLS function comprehensively necessitates the deployment of multi‐omics platforms that integrate whole‐exome sequencing, RNA‐seq, proteomics, metabolomics, and spatial omics [72, 123]. Fukuda et al. [72] have employed such an integrative approach to delineate the immune phenotypes of NSCLC molecular subtypes, thereby establishing a foundation for subsequent AI‐driven modeling. In the proximal inflammatory subtype, the number of tumor‐infiltrating leukocytes increased, and the immune response in the tumor microenvironment was activated, as indicated by high levels of tertiary lymphoid structures and high cytotoxic marker levels [72].

##### 6.2.1.1 AI and Machine Learning Models for Prognosis

(i) Unsupervised learning approaches (PCA, cluster analysis) facilitate the identification of pathways associated with TLS formation and maturation. (ii) Supervised learning methods (random forests, support vector machines, neural networks) enable the construction of predictive models incorporating TLS density, maturity, spatial distribution, and multi‐omics features to forecast pCR/MPR or disease‐free survival/overall survival [185, 186]. A multimodal foundation model trained on over 1000 NSCLC tumor tissues with paired spatial transcriptomics, multiplex immunofluorescence, and H&E imaging has demonstrated the ability to efficiently segment TLS and identify genes that promote TLS maturation [185]. Furthermore, unsupervised multi‐omics integration using frameworks such as MOFA2 has revealed latent molecular subtypes in NSCLC that capture divergent survival outcomes and biologically coherent processes [186].

##### 6.2.1.2 Challenges and Solutions

(i) Data standardization: batch effect correction via ComBat or analogous algorithms [187]. (ii) Sample size constraints: multi‐center collaborative initiatives [188]. (iii) Model interpretability: Shapley values, LIME, and related explainability frameworks [189, 190].

##### 6.2.1.3 Future Directions

Integration of spatial transcriptomics and multiplex immunofluorescence with AI‐based analytical pipelines to dissect intratumoral TLS heterogeneity [185]. Construction of spatiotemporal TLS evolution models through the convergence of dynamic multi‐omics and longitudinal imaging data [141, 179].

#### 6.2.2 Comparative Analysis of TLS Functional Heterogeneity Across Tumor Types

Although TLS are commonly identified across diverse solid tumor types, their cellular composition, spatial distribution, and immunoregulatory functions exhibit substantial inter‐tumoral variation [10, 53]. Large‐scale cross‐tumor comparative studies are therefore essential to inform tumor type‐specific immunotherapeutic strategy design [38].

##### 6.2.2.1 Tumor Type‐Specific Characteristics

In NSCLC, TLS are predominantly localized at the tumor periphery and peritumoral stroma, correlating with favorable prognosis and enhanced sensitivity to PD‐1/PD‐L1 inhibitors [42, 60, 191]. In breast cancer, particularly the triple‐negative subtype, TLS frequently co‐occur with elevated expression of immune‐related gene signatures, suggesting their potential utility in immune checkpoint inhibitor therapy [192]. In melanoma, TLS density positively correlates with the proportions of plasma cells and T follicular helper (Tfh) cells, influencing the inflammatory state of the tumor immune microenvironment and the maintenance of immunological memory [13].

##### 6.2.2.2 Cross‐Tumor Distribution of B(IR) Cell Subsets

A population of CD20^+^CD22^+^ADAM28^+^ memory B cells (termed BIR cells) has been identified across multiple solid tumor types, including cutaneous squamous cell carcinoma, renal cell carcinoma, colorectal cancer, and breast cancer. These cells are highly enriched within TLS and exhibit positive correlation with immune checkpoint inhibitor (ICI) efficacy [63]. Comparative analyses reveal that, although BIR cells are consistently localized within TLS across tumor types, their phenotypic features—including antigen‐presenting capacity and cytokine secretion profiles—vary substantially, likely reflecting tissue‐specific microenvironmental cues and tumor antigen characteristics.

##### 6.2.2.3 Implications for Personalized Therapeutic Strategies

Elucidation of TLS heterogeneity across tumor types can guide the rational design of TLS‐inducing agents (e.g., CXCL13 agonists) or TLS‐modulating interventions (e.g., targeted depletion of excessively immunosuppressive Tregs) to restore local immune equilibrium. Integration of multi‐omics and single‐cell technologies enables systematic cross‐tumor comparison of the association between TLS maturation status and immune effector cell activity, thereby facilitating the formulation of precision immunotherapy combination regimens tailored to distinct tumor types.

### 6.3 Clinical Trial Design and a Novel Paradigm for Individualized Precision Therapy

#### 6.3.1 Adaptive Neoadjuvant Therapy Trial Design Guided by TLS Scoring

A TLS‐centric scoring system can provide a quantitative framework for the individualized design and dynamic adjustment of neoadjuvant therapeutic regimens. The operational workflow encompasses the following components.

##### 6.3.1.1 Construction of the TLS Scoring System

Integration of TLS density (number per unit area), maturity (presence of germinal center structures and highly specific lineage markers), and spatial distribution (intratumoral versus peritumoral compartments) yields a multidimensional semi‐quantitative scoring metric. Digital pathology platforms (e.g., digital H&E and multiplex immunofluorescence image analysis) enable automated extraction of TLS parameters.

##### 6.3.1.2 Guiding Adaptive Therapeutic Adjustment

During neoadjuvant chemotherapy or chemoimmunotherapy, periodic reassessment (e.g., following every two treatment cycles) of TLS scores—derived from tumor tissue, ctDNA, or combined imaging modalities—can inform real‐time treatment decisions. A sustained increase in TLS scores or attainment of a predefined threshold may support continuation of the current therapeutic regimen; conversely, declining or suboptimal scores may warrant the timely introduction of additional immunotherapeutic agents or radiotherapy.

##### 6.3.1.3 Clinical Trial Case References

As Tang Xiaoping et al. [61] illustrated, the systemic immune‐inflammation index (SII) has been demonstrated to correlate positively with TLS abundance and maturity, supporting its utility as a non‐invasive TLS surrogate marker [66]. The platelet‐to‐lymphocyte ratio (PLR) similarly represents an independent predictor of high TLS expression; the combination of PLR with TLS scoring outperforms either parameter alone in predicting MPR rates following neoadjuvant chemoimmunotherapy in NSCLC [82].

##### 6.3.1.4 TLS‐Related Gene Risk Models

Shi‐Qi Mei et al. [80] employed weighted gene co‐expression network analysis (WGCNA), LASSO regression, and multivariate Cox proportional hazards modeling to construct a risk scoring model based on five TLS‐associated genes (RGS8, RUF4, HLA‐DQB2, THEMIS, TRBV12‐5). This model accurately predicts prognosis in NSCLC patients receiving immunotherapy and, when combined with clinical characteristics, demonstrates superior performance in predicting progression‐free survival (PFS) and overall survival (OS) compared with conventional clinical staging alone.

##### 6.3.1.5 Design of Individualized Treatment Regimens

For patients with high‐abundance, high‐maturity TLS, immune checkpoint inhibitors or low‐dose radiotherapy combined with immunotherapy may be prioritized to further potentiate TLS‐mediated immunity. For patients with low TLS expression, early introduction of CXCL13 agonists, low‐dose radiotherapy, or Treg‐targeted agents may promote TLS neogenesis and functional enhancement [37, 193, 194, 195]. Integration of metabolic modulation strategies (e.g., glycolysis inhibition in papillary‐pattern lung adenocarcinoma) and CAF‐targeting approaches may further remodel the tumor immune microenvironment and augment overall therapeutic efficacy [56].

Through the multi‐level research and clinical application framework delineated above, the integration of TLS into neoadjuvant therapeutic strategies and individualized precision medicine systems holds the potential not only to enhance treatment benefits for NSCLC patients but also to establish a paradigm for TLS‐directed immunotherapy across diverse solid tumor types.

## 7 Conclusions

### 7.1 The Central Role of TLS in Neoadjuvant Therapy for Lung Cancer

Systematic integration of multi‐omics data and clinical evidence has delineated the central role of tertiary lymphoid structures (TLS) in neoadjuvant therapy for lung cancer (Table 3).

**TABLE 3:** Clinical applications of TLS in NSCLC patients with neoadjuvant chemoimmunotherapy.

Tumor type	Markers	Characteristics of TLS	Year
NSCLC	CD3, CD20, CD21	The maturity of TLS may serve as a biomarker to improve the predictive accuracy of DFS in NSCLC patients receiving neoadjuvant chemotherapy and immunotherapy	2022 [60]
NSCLC	SII, PLR, NLR, LMR	Higher SII values show a negative correlation with TLS expression in the tumor microenvironment	2023 [66]
NSCLC	H&E	The appearance of follicular B cells (mainly IgM subtype) is associated with the formation of TLS and is located at the center of the TLS region	2025 [22]
NSCLC	CD8	The combination of neoadjuvant nivolumab, ipilimumab, and chemotherapy showed higher tumor immune cell infiltration and tertiary lymphoid structure compared to neoadjuvant nivolumab plus chemotherapy	2023 [196]
LUAC	CD20, CD21, CD23, PNAD, DC‐LAMP	The use of corticosteroids during chemotherapy negatively impacts the development of tertiary lymphoid structures and eliminates their prognostic value in lung cancer patients	2018 [20]
NSCLC	SII, PLR, NLR and LMR	PLR is an independent predictive factor for TLS, and both TLS and SII can predict the prognosis of resectable NSCLC patients after neoadjuvant chemotherapy immunotherapy; however, combining SII and TLS to assess DFS is more accurate than using either parameter alone	2023 [82]
LUAD, LUSC	The maturation, number and density of TLS	High TLS/TLS+ is associated with improved OS, DFS, RFS, and DSS, and TLS/TLS+ is associated with improved DFS in patients receiving immunotherapy	2025 [45]
NSCLC	H&E	Patients with PCR had significantly prolonged DFS, and those with high TLS expression and high maturity level showed better clinical benefits than those with low expression and low maturity	2023 [51]
NSCLC	CD163, CD68, PD‐1, PD‐L1, CD3, CD4, CD8, CD56, CD20, Foxp3, and pan‐cytokeratin (pan‐CK)	Patients with tertiary lymphoid structures (TLSs) showed a significant increase in the proportion of M1 macrophages and lymphocytes near tumor cells, while in patients without TLSs, PD‐L1+ tumor cells and PD‐1 + CD8+ T cells were more densely clustered together. Neoadjuvant immunochemotherapy induced the formation of TLS	2024 [197]
NSCLC	CD163, CD68, PD‐1, PD‐L1, CD3, CD4, CD8, CD56, CD20, Foxp3 and pan‐CK or S100	Higher numerical density of TLS was observed in the TIME of MPR patients. Moreover, MPR patients showed significantly higher infiltration of TLS, CD3+ cells, and CD20+ cells compared to those receiving chemotherapy	2022 [198]
Early‐Stage LUAD	CD3, CD4, CD8, CD19, CD68, CD56, FOXP3, CD163, PD1, PD‐L1, TIM‐3, LAG‐3	High levels of immunosuppressive molecules in tumors, lymph nodes and/or peripheral blood may indicate poor outcomes after immunotherapy, even in patients with both tuberculoid‐like responses and tertiary lymphoid structures	2022 [199]
NSCLC	PD‐L1, CD8	In this study on immune related biomarkers in locally advanced resectable NSCLC, CD8+ TILs and TLS were prognostic factors but did not yield additional information to age and TNM in multivariable analyses	2022 [71]
NSCLC	CD3, CD8, PD‐1, PD‐L1, CD68, FOXP3, granzyme B, CD45RO, pancytokeratin	Proof‐of‐concept ST analysis of a CPR HLA‐DEF tumor after ChIO showed a strong immune response with tertiary lymphoid structures (TLS), CD4+ T cells with HLA class II colocalization, and activated CD8+ T cells	2024 [200]
NSCLC	CD138, CD4	Localized B cell clusters associated with TLS formation, B‐cell activation and maturation were selectively observed in post‐treatment lung tissue along with sparse antibody‐producing plasma cells	2025 [201]
NSCLC	H&E	As the 14‐gene risk stratification increased, patients showed markedly decreased levels of TIL infiltration and TLS positivity rates. The low‐risk group exhibited significantly higher proportions of robust TIL infiltration (3+) compared to the high‐risk group, along with notably elevated TLS positivity rates	2025 [202]
NSCLC	CD3E, CD4, CD8A, CD19, PTPRC, CCL19, CCR7, CD28, LTA, CXCR3, IFNG, IL2, IL12B, IL15, TBX21, TNF, FASLG, GZMB, PRF1, CCL5, CCR2, CCR4, CCR5, CD40LG, CTLA4 and CSF2	Chemotherapy can induce the formation of TLS, and PD‐1 blockade can enhance the function of TLS	2024 [61]
LUSC	H&E	Formation of tertiary lymphoid structures after neo‐adjuvant immunotherapy: Tertiary lymphoid structures are commonly observed in tissues of cases with complete and significant pathological response, while their presence varies in tissues of cases with non‐significant pathological response	2021 [203]

As in situ immune organs, TLS orchestrate the spatial organization of B cells, T cells, dendritic cells, and additional immune effector populations, thereby establishing a platform for local anti‐tumor immune responses within the tumor microenvironment [204]. From an immunoregulatory perspective, TLS function as active immune factories during the neoadjuvant treatment phase: therapeutic intervention not only induces tumor cell apoptosis and necrosis, but also activates local immune compartments, promoting B cell maturation and tumor‐specific antibody production, which in turn facilitates the activation and maintenance of CD8^+^ cytotoxic T cells and tissue‐resident memory T cells [205, 206]. This self‐reinforcing anti‐tumor immune circuit mechanistically underpins the pathological complete response (pCR) or major pathological response (MPR) observed in a subset of patients following neoadjuvant immunotherapy, establishing a causal relationship between dynamic TLS remodeling and therapeutic response [60].

With respect to spatial distribution, TLS localized in the tumor periphery and core regions exhibit distinct immune surveillance functions. Peritumoral TLS display enhanced capacity for effector immune cell recruitment and activation, thereby exerting more potent anti‐tumor activity [53]. This spatial heterogeneity is further reflected in TLS maturation stratification: the cooperative functionality of immune cells within fully mature TLS, as contrasted with that within immature or primary‐stage structures, directly correlates with long‐term survival outcomes following neoadjuvant therapy. Consequently, multidimensional evaluation encompassing TLS density, maturity, and intratumoral spatial distribution constitutes a robust biomarker framework for treatment response and prognosis determination.

The neoadjuvant therapeutic window provides a unique opportunity for TLS induction and maturation. Distinct from conventional adjuvant or single‐modality chemotherapy/radiotherapy regimens, neoadjuvant therapy not only achieves direct tumor cell killing, but also—through the integration of immunotherapy, chemotherapy, and low‐dose radiotherapy to stimulate TLS formation and functional remodeling [12]. Low‐dose radiotherapy, in particular, has been demonstrated in experimental models to promote rapid TLS formation and enhance maturation, providing a mechanistic rationale for its incorporation as a TLS‐inducing modality in combinatorial regimens [74]. Concurrently, advances in multimodal immune analytical technologies—including multiplex immunofluorescence, spatial transcriptomics, and AI‐driven image analysis—have enabled precise quantification of TLS cellular composition and spatial architecture, further consolidating the status of TLS as predictive biomarkers with distinct advantages over conventional PD‐L1 expression and tumor mutational burden assessment systems.

In clinical practice, TLS detection and quantitative evaluation through pathological assessment (H&E staining, immunohistochemistry) combined with digital pathology approaches have yielded preliminary validation. Furthermore, composite scoring models integrating transcriptomic and immunomic data have demonstrated the capacity to statistically discriminate long‐term survivors from patients at elevated risk of early recurrence. These findings not only substantiate the core role of TLS in neoadjuvant therapy for lung cancer but also underscore their translational potential in future individualized precision treatment paradigms (Table 3). Integrating TLS‐based scoring systems into patient stratification and neoadjuvant regimen formulation enables a conceptual transition from single‐modality immunotherapy toward combined multimodal intervention—simultaneously targeting tumor cells and remodeling the local immune microenvironment to achieve superior anti‐tumor efficacy.

Collectively, TLS not only occupy a central position in driving anti‐tumor immune responses during neoadjuvant therapy for lung cancer, but also can hold critical significance for prognostic assessment and individualized treatment design (Table 3). By synthesizing clinicopathological parameters, molecular biomarkers, and neoadjuvant treatment responses, TLS catalyze a strategic paradigm shift from “tumor cell elimination alone” to “coordinated immune microenvironment remodeling,” thereby providing a robust theoretical and practical foundation for future precision cancer immunotherapy.

### 7.2 Current Research Limitations and Future Directions

Notwithstanding the substantial advances delineated above, a series of technical limitations and mechanistic questions remain unresolved.

#### 7.2.1 Technical Limitations

Current TLS assessment predominantly relies on static tissue biopsies obtained pre‐ and post‐treatment. This static detection modality is inherently limited in capturing the dynamic processes of TLS formation, maturation, and spatial remodeling, and is susceptible to sampling bias arising from intratumoral heterogeneity. The development of non‐invasive, real‐time dynamic monitoring technologies—such as PET‐based tracer imaging or ctDNA immune repertoire profiling—is therefore imperative to enable longitudinal tracking of TLS formation and maturation, thereby providing critical support for dynamic pathological assessment and timely precision treatment adjustment.

#### 7.2.2 Mechanistic Gaps in Immune Microenvironment Biology

Substantial knowledge gaps persist regarding intercellular interaction networks within TLS. In particular, the selective clonal expansion dynamics of distinct immune subsets within TLS and the mechanisms governing tumor neoantigen presentation remain incompletely elucidated. Current evidence suggests that regulatory T cells (Tregs) exert a dual role within TLS: while excessive Treg accumulation may impede the full‐scale development of anti‐tumor immune responses, a basal level of immunoregulation is essential for maintaining local immune homeostasis and preventing inflammatory tissue damage [207]. Elucidating how to selectively promote the efficient activation of effector immune cells (CD8^+^ T cells, tissue‐resident memory T cells) while constraining the deleterious effects of excessive immunosuppressive components represents a question of critical importance [53, 208]. In‐depth dissection of the spatiotemporal coupling between immune clonal evolution within TLS, the functional status of antigen‐presenting cells, and cross‐regulatory interactions with driver mutations (e.g., EGFR) will provide novel mechanistic insights and experimental foundations for future targeted regulatory strategies [54].

#### 7.2.3 Cross‐Cancer and Multi‐Center Validation

The majority of TLS studies to date have focused on single tumor types or localized treatment effect evaluation; cross‐cancer, multi‐center prospective cohort studies remain insufficient [159]. To establish TLS as mature predictive biomarkers and therapeutic targets in clinical practice, large‐scale, multi‐center prospective clinical trial designs should be prioritized [209]. Such designs will not only validate the relationship between TLS maturation and patient prognosis at scale, but also it can enable the exploration of adaptive neoadjuvant therapeutic regimens guided by TLS scoring. Following the TRACERx paradigm, longitudinal follow‐up with multi‐point sample collection could facilitate the construction of mathematical models linking TLS dynamics to immunotherapy response, thereby providing enhanced quantitative support for clinical decision‐making [159, 210].

#### 7.2.4 Metabolic and Molecular Characterization

Current research relies predominantly on histopathological and transcriptomic data; the metabolic landscape and local molecular regulatory networks within TLS remain comparatively underexplored [211]. Given that local metabolic parameters—including lactate concentration and glucose metabolic activity—profoundly influence immune cell function and are intimately linked to TLS formation and maturation [211], high‐resolution approaches integrating metabolomics, spatial transcriptomics, and single‐cell sequencing are urgently needed to dissect the mechanistic relationships between intratumoral metabolism, molecular regulation, and TLS biology, thereby revealing the internal mechanisms underlying currently poorly understood TLS dysregulation and local immune exhaustion [102].

#### 7.2.5 Clinical Translation

Although the close association between high TLS expression/maturation and prolonged survival following neoadjuvant therapy has been preliminarily validated, a series of technical and practical bottlenecks impede the rapid translation of TLS detection technologies into routine clinical diagnostics [210]. Key challenges include detection standardization, scoring system harmonization, and multi‐center data integration [159]. Future efforts should prioritize the construction of standardized TLS scoring frameworks that integrate pathological images, immunohistochemical markers, and digital pathology outputs [212]. Leveraging AI and deep learning technologies, a comprehensive suite of precise evaluation models should be developed to achieve rapid, non‐invasive TLS quantification in clinical settings, thereby furnishing a robust decision‐making platform for individualized treatment planning [210, 213]. Concurrently, clinical translation trials should be designed targeting distinct neoadjuvant regimens (immunotherapy alone, chemotherapy plus immunotherapy, low‐dose radiotherapy plus immunotherapy, etc.) to delineate the optimal matching between TLS dynamics and treatment timing, and to explore novel TLS‐modulating combination strategies [53].

#### 7.2.6 Long‐Term Vision

The ultimate objective of future TLS research is the realization of a closed‐loop therapeutic paradigm encompassing “TLS induction–monitoring–enhancement.” [214] Specifically: during the early phase of neoadjuvant therapy, exogenous factors (e.g., CXCL13 agonists, low‐dose radiotherapy) are deployed to directly promote de novo TLS formation [53, 159]; dynamic monitoring is conducted throughout the treatment course, with rapid therapeutic regimen adjustment via targeted intervention upon detection of declining TLS abundance or maturity; and through longitudinal follow‐up and iterative refinement, the conversion from immunologically “cold” to “hot” tumors is ultimately achieved, culminating in comprehensive local immune system activation for complete tumor eradication and recurrence prevention. This TLS‐centric, whole‐process intervention framework not only advances personalized precision treatment for lung cancer patients but also provides a paradigm for the management of other solid tumor types [159].

In conclusion, while a substantial body of evidence has established the core role of TLS in neoadjuvant therapy for lung cancer and has partly elucidated the relationship between their immunoregulatory mechanisms and clinical prognosis, the limitations of current technical approaches, the complexity of underlying immune mechanisms, and the practical challenges of multi‐center clinical translation remain critical obstacles to be surmounted [159]. With the continued advancement of multimodal technologies and artificial intelligence‐based analytical methods, more refined dissection and clinical application of TLS are anticipated [210]. It is foreseeable that TLS will transcend their current status as biomarkers to become key regulatory targets in precision cancer immunotherapy, catalyzing a fundamental paradigm innovation in treatment concepts during the neoadjuvant era for lung cancer [214].

#### 7.2.7 Near‐Term Priorities

Optimization of dynamic monitoring technologies and construction of standardized scoring systems [212, 213]. Medium‐ to long‐term objectives: multi‐center trials validating the role of TLS in multimodal therapy, identification of novel TLS drivers, and development of optimized combinatorial immunotherapy regimens [53, 214]. With the sustained deepening of clinical and basic research, TLS are poised to become indispensable biomarkers and therapeutic targets in neoadjuvant therapy for lung cancer, ultimately realizing closed‐loop management across the entire continuum from prediction through intervention to efficacy optimization [132, 159].

## Content

The authors have nothing to report.

## Author Contributions

**Ling Yi:** conceptualization, methodology, visualization, supervision. **Zhiqi Li:** conceptualization, formal analysis, writing – original draft, writing – review and editing. **Yujie Dong:** methodology, investigation, visualization, writing – review and editing. **Lina Zhang:** conceptualization, methodology, validation, visualization, writing – review and editing. **Yuanming Pan:** conceptualization, methodology, writing – review and editing, funding acquisition, supervision. **Weiying Li:** conceptualization, formal analysis, validation, investigation. **Jinghui Wang:** conceptualization, funding acquisition, visualization.

## Funding

Beijing Municipal Public Welfare Development and Reform Pilot Project for Medical Research Institutes (JYY2023‐14) to J.W. Beijing Traditional Chinese Medicine Science and Technology Development Fund Project (BJZYYB‐2025‐24); The Research Project of Inner Mongolia Medical University Affiliated Hospital (2023NYFYLHZD007), Beijing Municipal Administration of Hospital Incubating Program (PX2023059); The Capability Program of Beijing Tuberculosis & Thoracic Tumor Research Institute (NLTS2024‐16) to Y.P.

## Ethics Statement

This study did not involve human or animal subjects, and thus, no ethical approval was required. The study protocol adhered to the guidelines established by the journal.

## Conflicts of Interest

The authors declare no conflicts of interest.

## Data Availability

Data sharing not applicable to this article as no datasets were generated or analysed during the current study.
